# 3D Printing of Porous Ceramics for Enhanced Thermal Insulation Properties

**DOI:** 10.1002/advs.202412554

**Published:** 2024-12-25

**Authors:** He Lin, Qintao Shen, Ming Ma, Renquan Ji, Huijun Guo, Huan Qi, Wang Xing, Huiping Tang

**Affiliations:** ^1^ Advanced Materials Additive Manufacturing Innovation Research Centre College of Engineering Hangzhou City University Hangzhou 310015 P. R. China; ^2^ School of Mechanical Engineering Zhejiang University of Technology Hangzhou 310014 P. R. China; ^3^ Shenzhen Institute of Advanced Technology Chinese Academy of Sciences Shenzhen 518055 P. R. China

**Keywords:** 3D printing, artificial intelligence, pore structures, porous ceramics, thermal insulation

## Abstract

Porous thermal insulating ceramics play a pivotal role in both industrial processes and daily life by offering effective insulation solutions that reduce energy consumption, enhance building comfort, and contribute to the sustainability of industrial production. This review offers a comprehensive examination of porous thermal insulating ceramics produced by 3D printing, providing an in‐depth analysis of various 3D printing techniques and materials used to produce porous ceramics, detailing the fabrication processes, advantages, and limitations of these methods. Recent advances in 3D printed porous thermal insulating ceramics are thoroughly examined, with a particular focus on pore structure design and optimization strategies for high‐performance thermal insulation. This review also addresses the challenges and barriers to widespread adoption while highlighting future research directions and emerging trends poised to drive innovation. By showcasing the transformative potential of 3D printing in revolutionizing traditional porous ceramics manufacturing methods and enhancing thermal insulation performance, this review underscores the critical role of 3D printed porous ceramics in advancing thermal insulation technology.

## Graphical abstract

This review investigates 3D printed porous thermal insulating ceramics, focusing on fabrication techniques, material selection, porous structure design, and performance optimization. It provides recent advancements, challenges, and potential solutions while highlighting future research directions, emphasizing the transformative role of 3D printing in revolutionizing the production of porous ceramics and advancing their applications in thermal insulation.

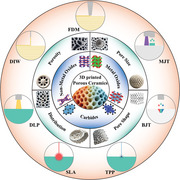

## 1 Introduction

With high‐temperature industrial procedures constituting a significant portion, ≈40%,^[^ ^1^, ^2^ ^]^ of global energy utilization, the imperative for the advancement of innovative strategies aimed at prudent energy usage and thermal conservation emerges.^[^ ^3^, ^4^ ^]^ One notable approach involves the incorporation of insulation materials to mitigate undesired thermal transfer, a trend increasingly pervasive across diverse industrial operations.^[^ ^5^, ^6^ ^]^ Thermal insulation materials comprise a diverse array of materials or composite configurations possessing distinct properties, including but not limited to low thermal conductivity, high porosity, lightweight, and flexibility.^[^ ^7^, ^8^, ^9^, ^10^ ^]^ These characteristics are meticulously engineered to obstruct, hinder, or counteract the thermal transfer between two regions characterized by disparate temperature gradients.

Ceramic materials are characterized by their non‐combustible nature, high resistance to elevated temperatures, and chemical corrosion, rendering them frequently utilized as thermal insulators.^[^ ^11^, ^12^ ^]^ Porous ceramics, in contrast to their dense counterparts, are characterized by a significant abundance of interconnected or closed pores. This feature imbues porous ceramics with several advantageous properties, including low density, high specific surface area, elevated toughness, remarkable thermal shock resistance, superior thermal insulation capabilities, heightened temperature stability, and a reduced dielectric constant, rendering them highly promising for diverse industrial applications.^[^ ^13^, ^14^, ^15^ ^]^ The structural attributes of porous ceramics, notably porosity, pore size, and pore wall thickness, play pivotal roles in determining their functional efficacy. Specifically, porosity and pore size govern the range of applicability of porous ceramics, whereas pore wall thickness influences their mechanical characteristics. Porous ceramics exhibit a broad spectrum of porosity ranging from 2.3% to 99% and pore sizes spanning from 3 nm to 3 mm.^[^ ^15^ ^]^ Varied pore structures endow porous ceramics with distinct functional applications. For example, closed‐pore porous ceramics are particularly advantageous for thermal insulation, as their ability to impede fluid flow, combined with high porosity, ensures excellent thermal insulation properties.^[^ ^16^, ^17^ ^]^ These attributes make porous ceramics widely applicable in high‐temperature insulation across diverse industries, including aerospace, construction, nuclear engineering, petrochemicals, and metallurgy.^[^ ^13^, ^18^ ^]^

Initially, the production of porous ceramics relied on high‐temperature ceramic powders that underwent molding, drying, and sintering processes.^[^ ^19^, ^20^ ^]^ However, this approach often faced challenges in achieving precise control over pore size and porosity, limiting the practical applicability of the resulting materials. Subsequently, the advent of innovative methodologies such as the sacrificial template approach, direct foaming technique, and 3D printing has significantly improved the adaptability and diversity of porous ceramic manufacturing, enabling precise control over pore size and porosity.^[^ ^21^, ^22^, ^23^ ^]^ Among these, 3D printing stands out as a transformative technology for the direct fabrication of porous ceramics. This advancement not only enhances manufacturing flexibility and efficiency but also addresses the limitations inherent in traditional molding techniques.

**Figure 1:**
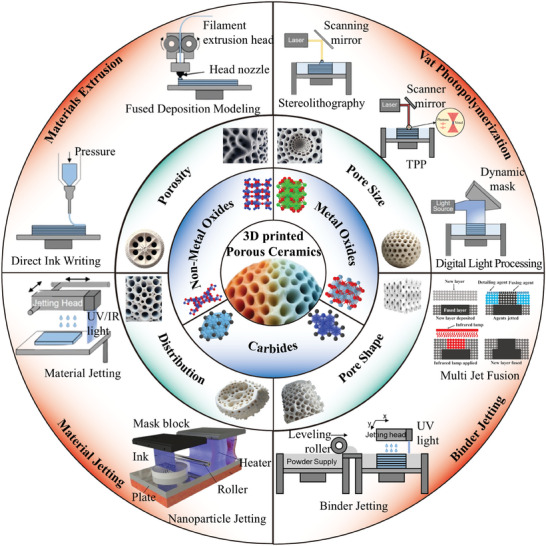
Schematic illustration showing the overall framework of the review. Reproduced with permission.^[^ ^24^, ^25^, ^26^ ^]^ Copyright 2019, Elsevier; Copyright 2021, Elsevier; Copyright 2022, MDPI.

The primary aim of this review is to furnish a comprehensive overview of 3D printing technologies utilized in the fabrication of porous ceramic materials tailored for augmented thermal insulation as shown in **Figure** **1**. Within this framework, an exhaustive examination of diverse 3D printing techniques and materials currently employed in porous ceramics production for thermal insulation is undertaken. It delves into methodologies for designing porous structures and explores optimization strategies to enhance thermal insulation performance. Additionally, the review identifies current challenges in 3D printed porous ceramics, highlights potential research directions, and investigates emerging trends. Ultimately, it underscores the pivotal role of 3D printed porous ceramics in advancing thermal insulation, emphasizing their transformative potential to revolutionize traditional insulation methods, enhance energy efficiency, and support sustainable development initiatives.

## 2 Fundamentals of Thermal Insulation

### 2.1 Definition and Principles of Thermal Insulation in Porous Materials

**Figure 2:**
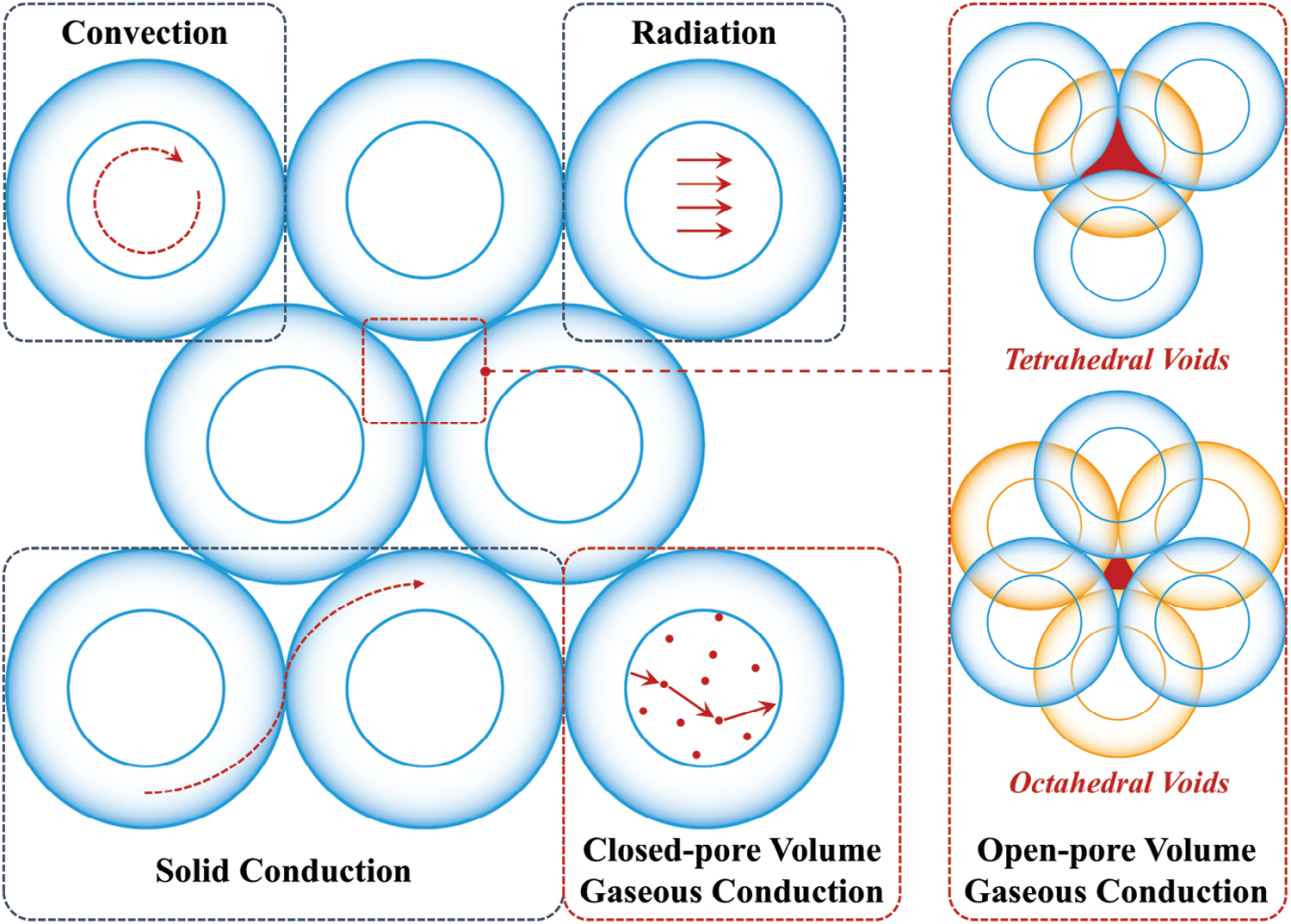
Thermal transport pathways in porous structures.

The thermal conductivity of porous materials is commonly described by their effective thermal conductivity, which integrates multiple heat transfer mechanisms, including solid conduction, gaseous conduction (within both open and closed pores), convection, and radiative transport (**Figure** **2**),^[^ ^27^, ^28^ ^]^ as shown in Equation (1).^[^ ^5^ ^]^

(1) $$\lambda_{\mathrm{eff}}=\lambda_{\mathrm{cond}}^{s}+\lambda_{\mathrm{cond}}^{g}+\lambda_{\mathrm{conv}}+\lambda_{\mathrm{rad}}$$

where *λ_conv_* represents thermal convection, it pertains to the large‐scale motion of fluids, including both liquids and gases, and signifies the transfer of thermal energy through fluid motion.^[^ ^29^ ^]^ In porous materials with pore sizes that shift from the micro‐to nano‐scale, the gas fluid becomes trapped by viscous forces, thereby completely suppressing gas convection and improving thermal insulation.^[^ ^30^ ^]^

*λ_rad_* (Thermal radiative transport) signifies the emission of electromagnetic waves from an object with a temperature exceeding absolute zero (0 K) during the heat transfer process.^[^ ^31^ ^]^ Unlike thermal conduction or convection, thermal radiative transport does not necessitate a medium for propagation and can occur even within a vacuum. The thermal conductivity of radiation can be expressed by Equation (2):^[^ ^30^ ^]^

(2) $$\lambda_{\mathrm{rad}}=\frac{16n^{2}\times \delta \times T^{3}}{3e\left(T\right)\times \rho }$$

where *δ* represents the Stefan‐Boltzmann constant, *n* denotes the average refractive index of the sample, *T* stands for the temperature, *e(T)* signifies the extinction coefficient expressed in m^2^/kg, and *ρ* corresponds to the apparent density of the porous material. This equation reveals that radiant thermal conductivity inversely correlates with the extinction coefficient, resulting in significant variations in thermal radiation behavior across materials with different extinction coefficients. Moreover, radiative thermal conductivity is directly proportional to the third power of the temperature, indicating that as the temperature increases, the amount of radiated heat increases substantially. This underscores the predominance of thermal radiation in heat transfer processes at elevated temperatures.

The primary and fundamental mechanism underlying thermal transfer is thermal conduction, primarily contingent upon the movement of microscopic energy carriers such as molecules, atoms, free electrons, and phonons.^[^ ^32^ ^]^ Solid thermal conduction ($\lambda_{cond}^{s}$) primarily hinges on the lattice vibrations of solid molecules in proximity to their equilibrium positions, whereas gas thermal conduction ($\lambda_{cond}^{g}$) originates from collisions among gas molecules.^[^ ^33^ ^]^ Thermal conduction in solids is contingent upon both the porosity of the material and the characteristics of the skeleton material.^[^ ^34^ ^]^ This relationship can be mathematically described by Equation (3).^[^ ^35^ ^]^

(3) $$\lambda_{\mathrm{cond}}^{s}=\lambda_{\mathrm{cond}}^{s,s}\;\frac{\rho \nu }{\rho_{s}\nu_{s}}$$

where $\lambda_{cond}^{s,s}$ represents the thermal conductivity of the skeleton, *ρ* and *ρ_s_* are the apparent density of the porous material and the true density of the skeleton, respectively; *ν* and *ν_s_* denote the speed of phonon in the porous material and skeleton, respectively. Increasing the material's porosity diminishes the effectiveness of solid heat transfer within the porous structure, thereby resulting in lower solid thermal conductivity. The thermal conductivity of the skeleton material is influenced by its intrinsic properties and crystal properties. In an ideal crystal, thermal resistance arises solely from phonon‐phonon scattering within the lattice. However, in actual materials, various crystal defects, such as vacancies, interstitial atoms, dislocations, interfaces, and grain boundaries, serve as sources of phonon scattering. These defects disrupt the phonon transport, leading to a reduction in thermal conductivity compared to that of a perfect crystal.^[^ ^36^, ^37^, ^38^ ^]^

Gas thermal conduction is the dominant mode of thermal exchange in most low‐density porous materials under ambient conditions.^[^ ^39^ ^]^ This conduction mechanism primarily depends on the pore size and the mean free path of air molecules within the porous structure, as well as the density and specific surface area of the material.^[^ ^40^ ^]^ The gas thermal conductivity in porous structures can be generally expressed by Equation (4):^[^ ^41^ ^]^

(4) $$\lambda_{\mathrm{cond}}^{g}=\frac{\lambda_{\mathrm{cond}}^{g,0}}{1+\alpha K_{n}}\;$$

where $\lambda_{cond}^{g,0}$ is the thermal conductivity of free gas, *α* is a constant related to gas species, *K_n_* is the Knudsen number, which is used to ascertain the continuity of the fluid flow. When *K_n_* >> 1, gas molecules primarily collide with the wall of the pore during their movement, making collisions between molecules themselves infrequent.^[^ ^42^ ^]^ Conversely, when *K_n_* << 1, gas behaves akin to a liquid flow state, with frequent collisions occurring between gas molecules.^[^ ^43^ ^]^ Clearly, the *K_n_* is intricately linked to the pore size of porous materials. In porous materials, *K_n_* can be determined by Equation (5):^[^ ^44^ ^]^

(5) $$K_{n}=\frac{l_{\mathrm{mfp}}}{l_{\mathrm{cl}}}$$

where *l_mfp_* denotes the average mean free path of gas molecules, which is influenced by the gas type and air pressure; *l_cl_* represents the average pore size of the porous material. Smaller average pore sizes correspond to larger *K_n_*, resulting in diminished gas thermal transfer efficacy.

### 2.2 Effect of Porous Ceramic Configuration on Thermal Insulation

As highlighted in the pervious section, the porosity, pore size, pore distribution, pore shape, and pore wall structure of porous thermal insulating ceramics constitute pivotal factors influencing thermal insulation performance. Careful regulation of these parameters enables precise tuning of the material's thermal conductivity, thereby optimizing its thermal insulation effectiveness. For example, porosity denotes the proportion of pore volume within a bulk material to its total volume in its natural state. Given the substantially lower thermal conductivity of gases compared to solids, an increase in porosity typically correlates with a decrease in material thermal conductivity.^[^ ^45^ ^]^ Pore size pertains to the dimensions of pores within a porous material. Smaller pores impede the thermal transfer process more effectively, as heat must traverse a greater solid‐gas interface area, resulting in an elongated thermal transfer path. Conversely, larger pores facilitate easier heat transfer through the material, potentially compromising thermal insulation performance.^[^ ^46^, ^47^ ^]^ Pore distribution refers to the spatial arrangement of pores within the material. Uniformly distributed pores disperse heat more evenly throughout the material, thereby reducing thermal transfer efficiency. Conversely, uneven pore distribution may lead to localized thermal transfer concentration, consequently affecting thermal insulation effectiveness.^[^ ^48^, ^49^ ^]^

The shape of pores influences both the length of the thermal transfer pathway and the characteristics of the thermal interface. For instance, spherical pores within porous materials result in shorter thermal transfer paths, whereas irregularly shaped pores may elongate the thermal transfer pathway.^[^ ^50^ ^]^ Furthermore, various shapes of pore interfaces exhibit distinct thermal resistances, consequently impacting the overall thermal insulation performance of the material.^[^ ^51^, ^52^ ^]^ The connectivity among pores within porous insulation materials directly influences the pathways of heat transfer within the material. When there is connectivity between pores, heat can swiftly propagate through these channels, resulting in an elevation of the overall thermal conductivity. Conversely, the presence of unconnected pores or constrained connectivity channels effectively diminishes the efficiency of thermal conductivity.^[^ ^14^, ^16^ ^]^

## 3 3D Printing for Porous Ceramics

### 3.1 Conventional Methods for the Fabrication of Porous Ceramics

**Figure 3:**
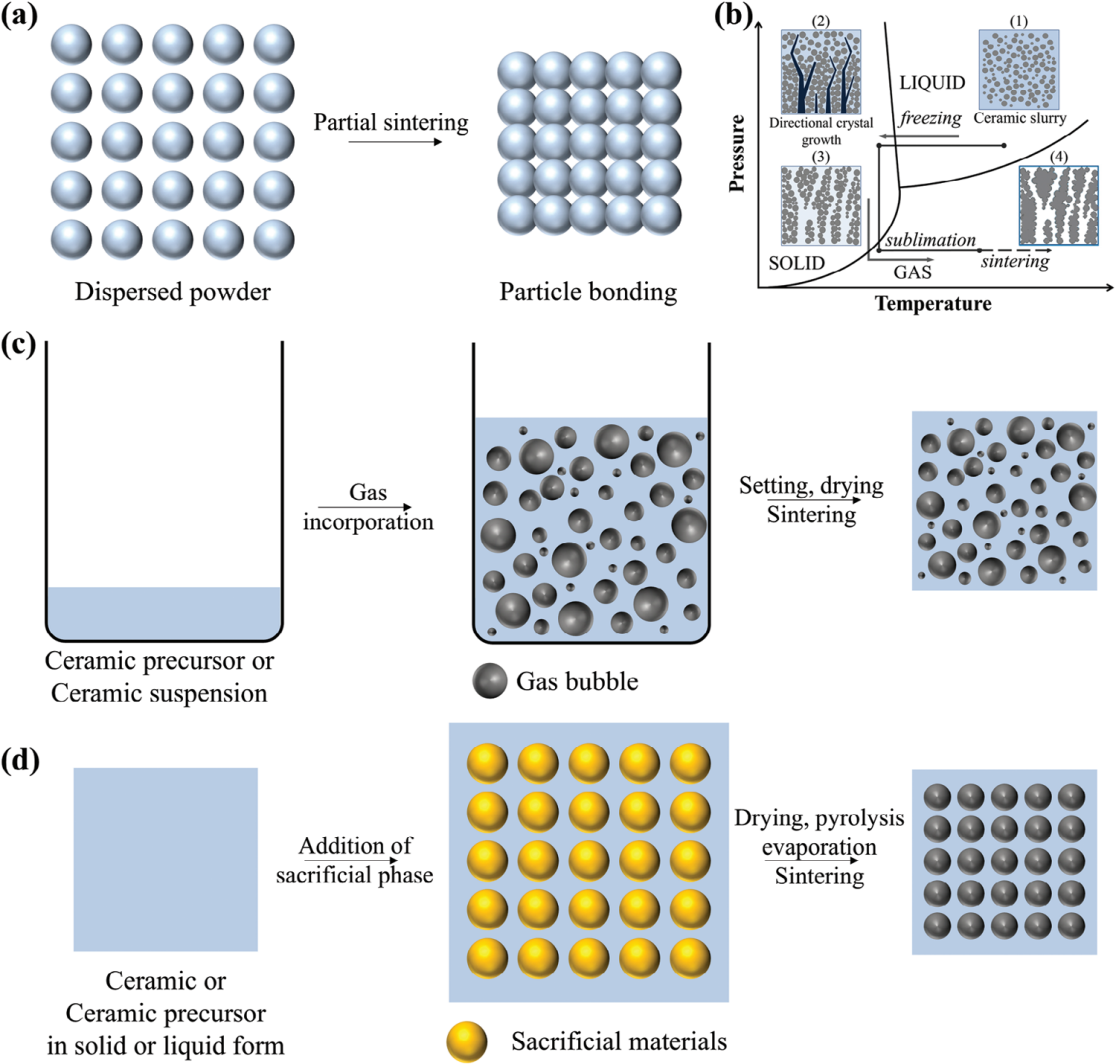
Schematic of a) partial sintering method and b) freeze‐drying method. Reproduced with permission.^[^ ^56^ ^]^ Copyright 2015, Elsevier. c) Direct foaming method and d) sacrificial template method for the production of porous ceramics.

The fabrication of porous ceramics necessitates precise control over their skeletal structures and pore architectures to fulfill specific application requirements. Conventional techniques, including partial sintering (**Figure** **3**a),^[^ ^53^, ^54^, ^55^ ^]^ freeze‐drying (Figure 3b),^[^ ^56^, ^57^, ^58^ ^]^ direct foaming (Figure 3c),^[^ ^59^, ^60^, ^61^ ^]^ and sacrificial templating (Figure 3d),^[^ ^21^, ^62^ ^]^ enable tailored porosity and pore morphology.^[^ ^63^ ^]^ However, each method is associated with inherent limitations, such as constrained control over pore uniformity,^[^ ^64^ ^]^ susceptibility to process variability,^[^ ^65^ ^]^ extended preparation durations,^[^ ^66^ ^]^ and elevated production costs.^[^ ^67^ ^]^ Challenges such as the thermodynamic instability of foams, the selection of suitable template materials, and potential compromises in pore integrity during processing underscore the complexities involved in achieving optimal performance in porous ceramics.^[^ ^68^, ^69^ ^]^

### 3.2 Overview of 3D printing for Porous Ceramic

The potential of 3D printing, also known as additive manufacturing, in the fabrication of porous ceramics is substantial, as it involves the direct computer‐aided design of intricate 3D structures for porous ceramics, followed by the physical realization of these designs through 3D printing, and subsequent refinement via post‐processing procedures tailored to the specific requirements of diverse 3D printing technologies.^[^ ^70^ ^]^ 3D printing enables precise control over pore geometry, size distribution, and interconnectivity, facilitating the synergistic optimization of insulation performance and mechanical strength tailored to specific applications.^[^ ^71^ ^]^ For example, 3D printing enables precise customization of porosity and pore size distribution by adjusting key parameters such as print path, layer thickness, and filling patterns, thereby optimizing the ceramic's thermal insulation properties.^[^ ^72^ ^]^ Additionally, this technology facilitates the creation of complex internal channels and structures that efficiently manage and control heat flow. For instance, strategically designed channel geometries can direct heat flow and minimize thermal bridging, significantly enhancing overall thermal insulation performance.

Furthermore, 3D printing supports the fabrication of multi‐material or functionally graded ceramics, enabling advanced features such as thermal gradient management and integrated mechanical enhancements within a single structure.^[^ ^73^ ^]^ Additional advantages include minimized material waste, reduced energy consumption, and accelerated prototyping cycles, contributing to more sustainable and cost‐effective production processes.

**Figure 4:**
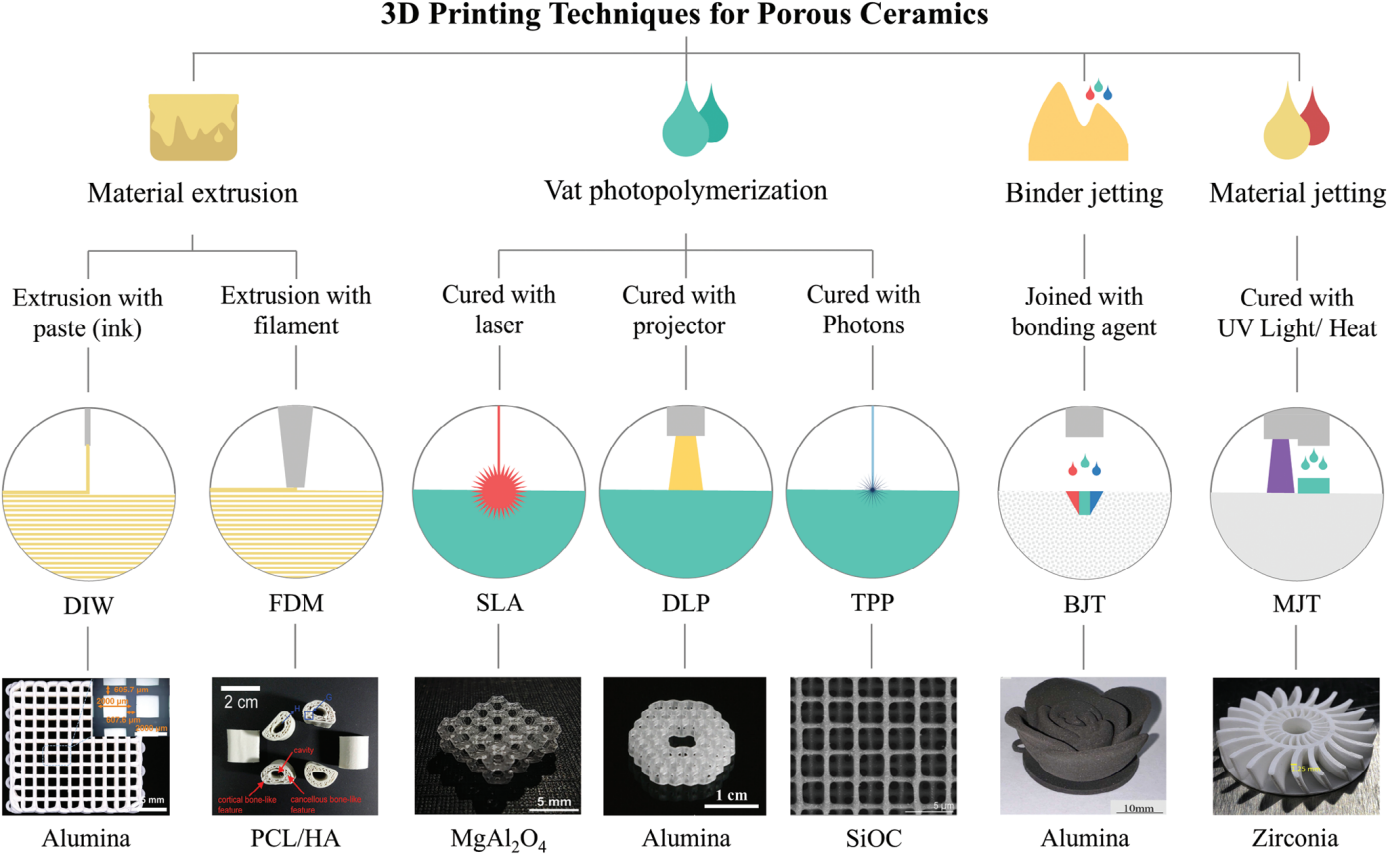
3D printing techniques used for fabricating porous ceramics. Reproduced with permission.^[^ ^24^, ^88^, ^89^, ^90^, ^91^, ^92^, ^93^ ^]^ Copyright 2023, Springer Nature; Copyright 2014, American Chemical Society; Copyright 2021, John Wiley and Sons; Copyright 2021, John Wiley and Sons; Copyright 2019, Elsevier; Copyright 2024, Elsevier; and Copyright 2019, Elsevier.

Following the classification established by the International Standards Organization (ISO) for 3D printing technologies, the methodologies deemed suitable for ceramic 3D printing are denoted as material extrusion (e.g., direct ink writing (DIW) and fused deposition modeling (FDM)),^[^ ^74^, ^75^, ^76^ ^]^ vat photopolymerization (e.g., stereolithography appearance (SLA), digital light processing (DLP) and two‐photon polymerization (TPP)),^[^ ^77^, ^78^, ^79^, ^80^, ^81^ ^]^ binder jetting (BJT),^[^ ^82^, ^83^, ^84^ ^]^ and material jetting (MJT)^[^ ^85^, ^86^, ^87^ ^]^ (**Figure** **4**). Each 3D printing technology features a distinct molding process, feedstock type, and product performance. The resolution of each technique varies significantly, with the printing process dictating the minimum achievable feature size, which subsequently determines the pore size of the fabricated porous ceramics. **Table** **1** summarizes the key parameters of representative 3D printing methods commonly utilized in the production of porous ceramics.

**Table 1:** Overview of the accuracy and forming dimension associated with typical 3D printing technologies for porous ceramics.

3D Printing Technologies	Accuracy	Forming Dimension	Refs.
Material extrusion	DIW: >100 µm	≈150 × 150 × 140 mm	[94]
	FDM: >100 µm	≈300 × 250 × 200 mm	[95]
Vat photopolymerization	SLA: 10–100 µm	≈320 × 320 × 200 mm	[96]
	DLP: 5–50 µm	≈180 × 180 × 100 mm	[97]
	TPP: <1 µm	≈100 × 100 × 8 mm	[98]
Binder jetting	> 100 µm	≈160 × 65 × 65 mm	[99]
Material jetting	≈20 µm	≈500 × 280 × 200 mm	[85]

Notably, the integration of 3D printing with conventional methods for preparing porous ceramics enables the fabrication of hierarchical porous structural ceramics, broadening the possibilities for designing materials with enhanced structural complexity and functional versatility. For instance, 3D printing can produce micrometer‐ and millimeter‐scale pore structures, while conventional freeze‐drying techniques effectively create nanometer‐scale pores. This combination facilitates the mass production of hierarchical porous ceramics with adjustable pore sizes. Such materials can exhibit not only high porosity and excellent thermal insulation properties but also remarkable mechanical strength.

### 3.3 Optimization of Pore Structure in 3D Printed Porous Ceramics

The optimization of the pore structure in 3D printed porous ceramics plays a pivotal role in enhancing thermal insulation properties. Well‐designed pore structures enable porous ceramics to act as effective thermal insulators by entrapping air or gases within the pores, thereby minimizing heat transfer via conduction and convection. Through precise manipulation of pore size, distribution, and interconnectivity, tailored materials with customized thermal conductivity can be fabricated, ensuring efficient insulation tailored to specific temperature ranges and environmental conditions.^[^ ^100^ ^]^ This is particularly crucial for applications demanding high‐temperature resistance, lightweight design, and enhanced energy efficiency.

The optimization process entails several key steps and considerations. First, the meticulous design of the 3D model is imperative, incorporating parameters such as pore size, shape, distribution, and interconnectivity to achieve desired properties. The selection of ceramic materials and fine‐tuning of printing technologies and parameters (e.g., temperature, layer thickness, and printing speed) are essential to control pore formation during the printing process. Post‐processing such as sintering and surface/interface treatment can further refine the pore structure to enhance thermal insulation performance. Lastly, comprehensive characterization methods including microscopy and pore structure analysis are employed to validate the suitability of the optimized pore structure for the intended thermal insulation application.

Computational modeling and simulation are indispensable tools for optimizing the design of porous structures in 3D‐printed porous thermal insulating ceramics. Techniques such as Finite Element Analysis and Computational Fluid Dynamics enable the simulation of thermal transfer processes within porous ceramic materials featuring diverse pore structures.^[^ ^101^ ^]^ These simulations enable the prediction of key properties, including thermal conductivity, heat storage capacity, and temperature distribution, under varying operating conditions. By leveraging these insights, the pore structure can be refined to achieve optimal thermal insulation performance. Iterative design improvements guided by simulation results enable the efficient determination of configurations that minimize heat transfer through conduction and convection, thereby enhancing thermal insulation efficacy.

By leveraging extensive datasets and advanced algorithms, Artificial Intelligence (AI) is set to revolutionize the optimization of pore structures in porous thermal insulation ceramics.^[^ ^102^ ^]^ Utilizing machine learning techniques, AI can identify complex correlations between pore structure, material properties, and thermal insulation performance, enabling pattern recognition and design refinement with unprecedented speed and precision. Through automated exploration of vast design spaces and virtual experimentation via computational simulations, AI‐driven design platforms can rapidly generate and evaluate multiple pore configurations, streamlining the selection of those with superior insulation capabilities. For example, Liu et al.^[^ ^103^ ^]^ introduced a conventional convolutional neural network (CNN) architecture incorporating the self‐attention and multiscale feature‐fusion mechanism, thereby enhancing the accuracy and stability of predicting the effective thermal conductivity (ETC) of porous media. The model's efficacy is visually demonstrated through gradient‐weighted class activation mapping (Grad‐CAM) of ETC, highlighting its superior predictive performance. Additionally, leveraging the quartet structure generation set (QSGS), a dataset comprising 10000 images of gradient porous ceramic media is constructed for training the CNN model. Comparative analysis of ETC prediction results and relative error distributions across various models reveal notable enhancements, including a 33.7% reduction in mean error, a 25.2% reduction in median error, and a substantial 59.6% reduction in maximum error. These results, combined with the enhanced Grad‐CAM visualizations, underscore the significant advancements achieved by the proposed model in accurately and reliably predicting ETC for ceramic materials with gradient porosity distributions. Moreover, AI enables the incorporation of practical considerations, such as manufacturing limitations, cost constraints, and environmental impact, into the design process. This ensures that the optimized pore structures are not only highly effective but also feasible and sustainable for real‐world applications.

In addition, the 3D printing process is critical to the fabrication of porous thermal insulation ceramics. In‐depth exploration of this optimization involves a comprehensive analysis of factors such as printing temperature, layer thickness, printing speed and nozzle diameter (for some 3D printing technologies, such as DIW, etc.), aiming to attain precise control over pore formation and distribution within the ceramic matrix. For instance, in the DIW process, fine‐tuning the printing temperature affects the viscosity and flow characteristics of the ceramic slurry, shaping the deposition pattern and influencing pore development during printing.^[^ ^104^ ^]^ Additionally, variations in nozzle diameter and layer thickness directly impact the resolution and accuracy of printed layers, consequently influencing pore size and interconnectivity.^[^ ^105^, ^106^ ^]^ Moreover, optimizing printing speed plays a crucial role in facilitating proper fusion between deposited layers, minimizing imperfections, and enhancing structural integrity while preserving the desired porosity levels.^[^ ^107^ ^]^ This intricate orchestration of process parameters not only governs the thermal conductivity and insulation efficacy of the resultant ceramics but also dictates fundamental properties such as mechanical strength, permeability, and durability, pivotal for their performance in demanding thermal insulation applications.

## 4 3D Printed Porous Ceramics for Thermal Insulation

Porous insulation ceramics encompass a range of materials, including metal oxides (e.g., Al_2_O_3_, ZrO_2_), non‐metallic oxides (e.g., SiO_2_), carbides (e.g., SiC), and specific composites (e.g., Al_2_SiO_5_, ZrSiO_4_). These materials are characterized by low thermal conductivity, excellent refractoriness, and high corrosion resistance, with porosities reaching up to 99% and densities below 10 mg cm^−3^, making them highly suitable for thermal insulation applications.^[^ ^108^ ^]^ Among these, composite ceramics such as Al_2_SiO_5_ and ZrSiO_4_ exhibit notable thermal insulating properties; however, their fabrication via 3D printing methods remains uncommon, rendering them outside the scope of this review. **Table** **2** shows the pore parameters and thermal conductivity of some typical 3D printed porous insulation ceramics. These ceramics offer tremendous potential for insulation applications in lunar and polar bases, battery systems in cold climates, civil engineering projects, and for protecting spacesuits, fire‐resistant suits, furnaces, and spacecraft operating under high‐temperature conditions.^[^ ^108^ ^]^

**Table 2:** Pore parameters and thermal conductivity of 3D printed porous thermal insulating ceramics.

Ceramic materials	3D Printing Technologies	Pore size [µm]	Porosity [%]	Thermal conductivity [W m^−1^ K^−1^]	Refs.
Al_2_O_3_	DIW	41.5	83.1	0.3	[109]
3Y‐TZP (TPMS structure)	DLP	1000 (Neighboring periods distance)	67	0.93 ± 0.035	[110]
Silica aerogels	DIW	0.0126 (average)	≈95	0.0159	[111]
Silica aerogels	DIW	0.002–0.2 (0.0377, average)	≈89.2	0.03243	[112]
Silivoxels	SLA	0.008–0.011	≈80	0.0191	[113]
	DIW	11			
Polyorganosiloxane‐based aerogels	DIW	0.002–0.1	–	0.03748	[114]
Silica aerogels	DIW	0.0662 (average)	≈90	0.03439	[115]
Alumina‐silica aerogels		0.0254 (average)	≈91	0.04334	
Titania‐silica aerogels		0.046 (average)	≈90	0.0326	
Fiber‐based porous Al_2_O_3_‐SiO_2_ ceramics	DLP	≈10–100	82	0.11	[116]
Polyimide‐silica aerogels	DIW	≈0.03 (average)	94	0.0208	[117]
Clay‐based ceramic	DIW	10–500 (large scales), <1 (small scales)	93–97	0.037–0.044	[118]
SiC	DLP	0.002–0.3	72–85	0.062–0.088	[119]
SiC (graded TPMS‐lattice structures)	DLP	≈229 (Neighbouring periods distance)	≈65	≈2	[12]
SiC	Binder jetting	≈50	≈44	≈15	[120]
SiC sandwich panels	DLP	100–160	87–90	0.049–0.057	[121]
SiC nanowire aerogels	DIW	≈0.02–200	94	0.046	[122]
Mullite‐reinforced SiC aerogels	DIW	<1∼≈10	90	0.021	[123]
Silicon oxycarbide ceramics	SLA	21 or 51 (wall distance)	≈76	0.12	[124]

In practical applications, the mechanical properties of porous thermal insulation ceramics are critical and are significantly influenced by key pore structure characteristics associated with high thermal insulation performance, such as high porosity, small pore size, uniform pore distribution, irregular pore shape, and closed pores.^[^ ^15^ ^]^ High porosity reduces material density, thereby increasing specific strength and stiffness, but often compromises absolute strength, whereas small pore sizes enhance internal friction and interlocking, improving mechanical properties. Uniform pore distribution minimizes stress concentrations, promoting isotropy and overall mechanical strength, while irregular pore shapes and uneven pore distributions can introduce defects and reduce toughness. Closed pores, however, enhance compressive strength by restricting deformation under pressure. These characteristics underscore the inherent trade‐off between thermal insulation performance and mechanical strength. 3D printing offers a promising solution by enabling the precise fabrication of complex structures and allowing for customized pore designs, such as small and uniform pores, graded porosity, or layered structures, to optimize thermal insulation performance without compromising strength. Advanced modeling techniques, including topology optimization and AI‐driven simulations, further support the design of innovative porous structures. Additionally, material innovations–such as ceramic‐polymer composites, functional additives like graphene, and protective coatings–contribute to enhanced overall performance.

Additionally, post‐processing, especially sintering, is a pivotal step in determining the final properties of 3D printed porous ceramics.^[^ ^19^, ^20^ ^]^ Due to their unique internal structures, layer interfaces, and porosity, these ceramics exhibit significantly different sintering behaviors compared to conventional solid ceramics, affecting densification, shrinkage, and mechanical stability. The complex geometries and porosities of porous ceramics often result in non‐uniform densification and internal stresses during sintering. Additionally, weak bonding at layer interfaces–a characteristic of the layer‐by‐layer 3D printing process–can lead to anisotropic shrinkage and reduced mechanical strength.^[^ ^82^ ^]^ While the deliberate introduction of porosity enhances functional properties such as thermal insulation and permeability, it also alters the sintering dynamics, frequently causing inhomogeneous shrinkage and localized deformation. Addressing these challenges requires precise control of sintering temperature and duration to maintain the designed pore structures and ensure dimensional accuracy. Furthermore, optimizing pore geometry, improving interlayer bonding, and fine‐tuning the sintering process can enhance the mechanical properties of porous ceramics while preserving their functional porosity.

### 4.1 Metal Oxides

#### 4.1.1 Alumina (Al_2_O_3_)

**Figure 5:**
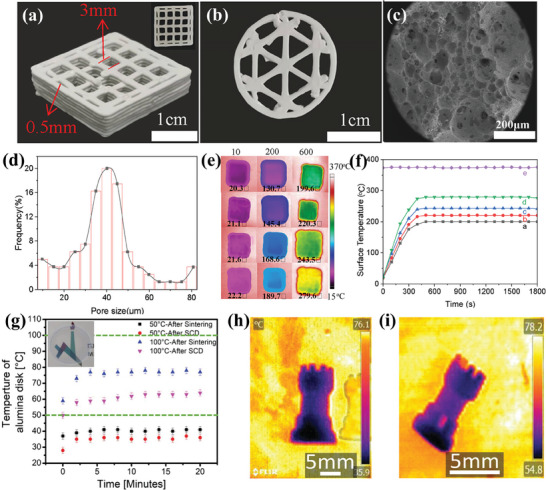
3D Printed‐Porous Al_2_O_3_ Ceramics for Thermal Insulation. a,b) 3D printing gel foams with various lattice shapes, c) SEM images of filament, d) size distribution of micrometer‐scale pores in 3D‐printed Al_2_O_3_ foams, e) infrared thermal images, and, f) surface temperature of 3D‐printed ceramic with different average sizes. Reproduced with permission.^[^ ^109^ ^]^ Copyright 2022, American Chemical Society. g) Time‐dependent behavior of the thermal insulation properties of the 3D printed alumina structures; Infrared image of the printed objects after (h) supercritical drying and (i) after sintering. Reproduced with permission.^[^ ^125^ ^]^ Copyright 2023, John Wiley and Sons.

3D printed porous Al_2_O_3_ ceramics exhibit a spectrum of outstanding characteristics, including superior thermal insulation, high tolerance to elevated temperatures, and exceptional corrosion resistance. As a result of these properties, they find extensive applications as thermal insulation materials across diverse fields such as high‐end chips, aerospace engineering devices, and fuel cells, etc. Yang et al.^[^ ^109^ ^]^ developed hierarchical porous alumina ceramics utilizing the DIW process (**Figure** **5**a–c), featuring pores ranging from millimeter to nanometer scales (Figure 5d). By adjusting the ammonium oleate content in the supramolecular gel, the micrometer‐scale pore sizes were successfully controlled, ranging from 41.5 to 137.4 µm. The resulting material exhibited excellent thermal insulation properties. For instance, the surface temperature of a 3D‐printed ceramic foam with 41.5 µm pore size was only 220.3 °C when placed on a hot plate at 380 °C, compared to a surface temperature exceeding 279.6 °C without ceramic foam protection (Figure 5e,f). Additionally, this ceramic foam proved effective for thermal management in electronic devices, such as circuit boards, where it reduced localized temperatures from 54.8 to 34.2 °C. This strategy highlights a novel approach to efficient thermal management for portable devices and consumer electronics by precisely regulating pore sizes to enhance thermal insulation performance. Moshkovitz et al.^[^ ^125^ ^]^ utilized a combination of DLP and sol‐gel reactions to fabricate transparent and porous γ‐alumina structures. These structures exhibited a high specific surface area exceeding 1800 m^2^ g^−1^ and a transmittance greater than 80% at 600 nm. In thermal insulation tests, the upper surface temperatures of the supercritical dried samples were lower than those of the sintered samples after 20 min of exposure to heat sources at 50 and 100 °C (Figure 5g–i). This superior thermal insulation performance is attributed to the higher air content in the supercritical dried samples, emphasizing the potential of supercritical drying in producing porous structures with enhanced thermal insulation capabilities.

#### 4.1.2 Zirconia (ZrO_2_)

**Figure 6:**
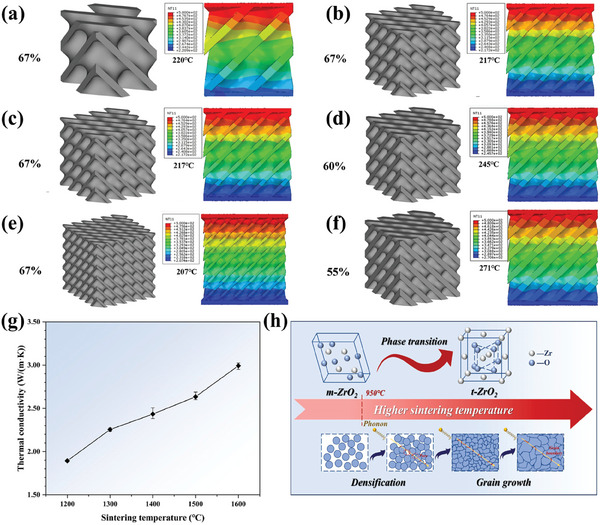
3D Printed‐Porous ZrO_2_ Ceramics for Thermal Insulation. a–f) 3Y‐TZP via DLP, thermal insulation simulations of TPMS‐type 3Y‐TZP structural ceramics. Reproduced with permission.^[^ ^110^ ^]^ Copyright 2023, Springer. g) The thermal conductivity of the YSZ ceramics at different sintering temperatures and h) mechanism of sintering temperature on the thermal conductivity. Reproduced with permission.^[^ ^126^ ^]^ Copyright 2023, Elsevier.

Porous 3 mol% yttria‐stabilized tetragonal zirconia polycrystal ceramics (3Y‐TZP ceramics) have garnered significant interest across automotive, marine, aerospace, and biomedical sectors owing to their distinctive attributes, including high‐temperature resilience, corrosion resistance, wear resistance, and low thermal conductivity. Yang et al.^[^ ^110^ ^]^ utilized DLP to fabricate fine Triply Periodic Minimum Surface (TPMS) structure blanks from a blend of 3Y‐TZP ceramic powder and photosensitive resin. Notably, diamond‐structured TPMS 3Y‐TZP ceramics achieved an exceptionally low thermal conductivity of 0.93 ± 0.035 W m^−1^ K^−1^ and a high compressive strength of 166.5 ± 27.3 MPa at 67% porosity (**Figure** **6**a–f). This work highlights an excellent balance between mechanical strength and thermal insulation properties, achieved through the strategic use of low thermal conductivity 3Y‐TZP ceramic materials. By leveraging a precisely controlled TPMS structural design and an optimized two‐step sintering process, the thermal conductivity was effectively reduced by tailoring the pore structure and porosity, while preserving the material's high strength. This integrated approach exemplifies a successful synergy between mechanical robustness and thermal insulation performance. The 3D printing and molding process of ceramic materials inherently involves sintering. Liu et al.^[^ ^126^ ^]^ reported that the thermal conductivity of yttria‐stabilized zirconia (YSZ) ceramics increased from 1.89 to 2.99 W m^−1^ K^−1^ as the sintering temperature rose, accompanied by a reduction in porosity from 15.00% to 1.59% (Figure 6g) and an increase in grain size from 0.13 ± 0.03 to 0.70 ± 0.20 µm. At lower sintering temperatures, the high porosity led to reduced thermal conductivity due to enhanced phonon scattering. However, at higher temperatures, particularly at 1600 °C, grain size became the dominant factor. The reduction in grain boundaries, caused by grain growth, minimized phonon scattering and thereby increased the thermal conductivity (Figure 6h).

Moreover, beyond individual metal oxide ceramics, combinations involving two or more metal oxide ceramics present promising opportunities for insulation applications, with 3D printing proving adept at accommodating such multi‐material compositions.^[^ ^127^ ^]^ For example, Zhang et al.^[^ ^128^ ^]^ demonstrated the high‐temperature resistance and thermal insulation properties of porous ZrO_2_‐Al_2_O_3_ ceramics, capable of withstanding temperatures up to 1300 °C with a low thermal conductivity of 0.0322 W m^−1^ K^−1^. While the porous ZrO_2_‐Al_2_O_3_ ceramics discussed in this study were not crafted via 3D printing, instances of such ceramics fabricated through 3D printing exist, as demonstrated by Wu et al.^[^ ^129^ ^]^ utilizing DIW technology and Jiao et al.^[^ ^130^ ^]^ employing DLP technology, both yielding porous ZrO_2_‐Al_2_O_3_ ceramics. Hence, the untapped potential for producing porous thermal insulating ceramic materials using 3D printing techniques persists, with ample prior research offering valuable insights and avenues for exploration in this burgeoning domain.

### 4.2 Nonmetal Oxides and Carbides

#### 4.2.1 Silica (SiO_2_)

**Figure 7:**
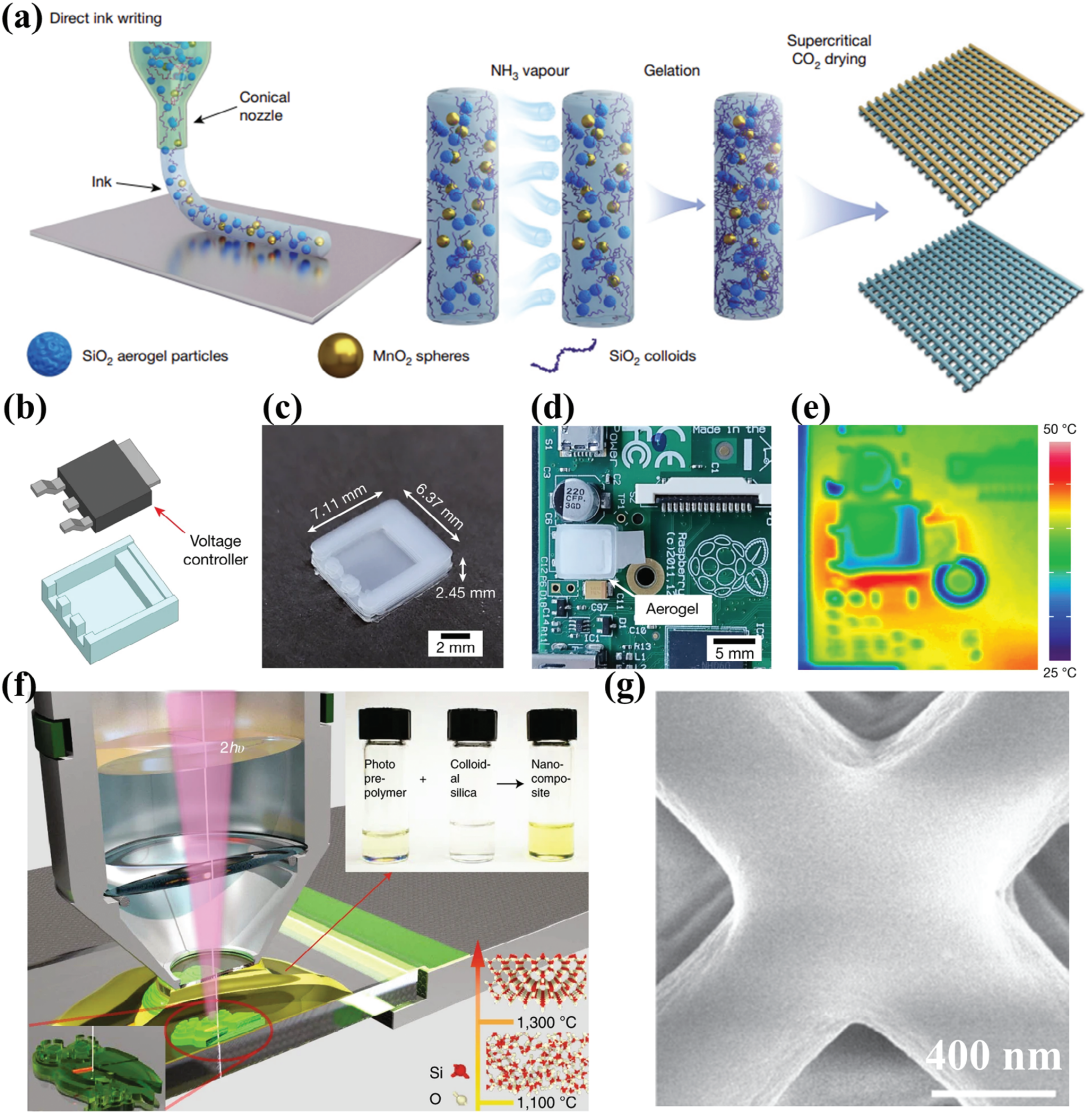
3D Printed Silica for Thermal Insulation. a) Silica aerogels via DIW, b‐e) pictures of a printed aerogel insulator cap to mitigate localized hot spots on the circuit board. Reproduced with permission.^[^ ^111^ ^]^ Copyright 2023, Springer Nature. f) Schematic of TPP‐enabled 3D‐printing set‐up and printing process, g) SEM images of 3D printed lattice structure. Reproduced with permission.^[^ ^131^ ^]^ Copyright 2021, Springer Nature.

Silica aerogel holds significant prominence in thermal insulation applications owing to its exceptionally low thermal conductivity and flexible pore structure, rendering it an ideal material for space‐constrained scenarios. However, its inherent brittleness poses a significant drawback. While fiber reinforcements and adhesives can mitigate this issue in various construction and industrial insulation contexts, their poor machinability and challenges in accurately casting small objects constrain the potential for miniaturizing silica aerogels. To address this limitation, Zhao et al.^[^ ^111^ ^]^ proposed a DIW approach for crafting miniaturized silica aerogels (**Figure** **7**a). This method involves formulating an ink by incorporating silica aerogel powder into a silica nanoparticle sol to create a suspension. Due to the high‐volume fraction of aerogel particles, the ink exhibits shear‐thinning behavior, facilitating fluidity during nozzle passage in the printing process, while swiftly increasing viscosity post‐printing to uphold the object's morphology. Subsequently, the printed structure undergoes further gelation and processing in an ammonia environment to yield an aerogel. The ultra‐low thermal conductivity of the printed silicone aerogel, measured at 15.9 mW m^−1^ k^−1^), is significantly lower than that of air (≈26 mW m^−1^ k^−1^) and conventional thermal insulating materials (typically exceeding 30 mW m^−1^ k^−1^). This exceptional property makes silicone aerogel an ideal material for space‐constrained applications, effectively limiting heat transfer. For instance, a tiny capacitor protected with a silicone aerogel cover maintains a localized temperature of just 36 °C, compared to 75 °C without protection and 48 °C with the same thickness of conventional insulating material (Figure 7b–e). Furthermore, the insulation performance can be tailored by varying the thickness of the printed silicone aerogel, enabling precise adaptation to diverse thermal management requirements. Additionally, the printed silicone aerogel membranes, with a Knudsen number (Kn) of 2.04, demonstrate potential for use in heat transfer gas pumps. By leveraging a thermal gradient, it can generate gas flow, making it highly suitable for micro‐gas pump applications. An et al.^[^ ^113^ ^]^ successfully integrated additive manufacturing with hierarchical assembly through multifunctional orthogonal surface hybridization of porous Silivoxels with polymer additives, resulting in exceptional machinability and structural controllability. Porous ceramics produced using this approach exhibit desired thermal insulation and mechanical properties, maintaining a low thermal conductivity of 19.1 mW m^−1^ k^−1^, a flexible compression recovery strain of 85%, and customized mechanical strengths ranging from 0.2 to 1.4 MPa.

Further pore size reduction to the nanoscale presents an avenue for manipulating thermal transfer within porous structures within the mean free path of air particles (Knudsen effect), offering promising prospects for various thermal management strategies. Wen et al.^[^ ^131^ ^]^ introduced a method for printing silica nanostructures using TPP, utilizing “inks” composed of poly(ethylene glycol)‐functionalized colloidal silicon nanoparticles and two‐photon polymerizable precursors. The TPP technique achieved the fabrication of porous ceramics with a resolution of less than 200 nm (Figure 7f,g). This method demonstrates the capability to reduce the pore size in 3D printed porous ceramics to the nanometer scale, which could significantly enhance thermal insulation performance.

Nevertheless, the extended service temperature of silica, particularly aerogels, is typically regarded as not surpassing 650 °C, as its porous framework tends to collapse beyond 600 °C.^[^ ^132^ ^]^ To enhance the thermal stability of silica, especially aerogels, researchers commonly employ doping or incorporating additional constituents.^[^ ^133^, ^134^, ^135^ ^]^ Leveraging the high melting point and stable crystal structure of alumina oxide, researchers blend alumina with silicon oxide to enhance temperature resistance and high‐temperature insulation properties.

**Figure 8:**
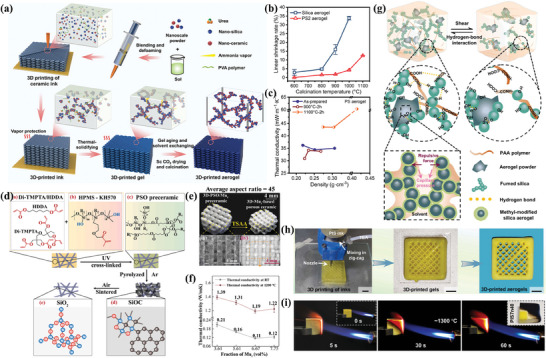
a) The thermal‐solidifying DIW to fabricate ceramic aerogels, b) linear shrinkage of aerogel with different treatment temperatures, and c) thermal conductivity versus density for 3D printed aerogels. Reproduced with permission.^[^ ^115^ ^]^ Copyright 2022, John Wiley and Sons. d) The phase transformation of HPMS‐KH570 during 3D printing, e) optical images, and f) thermal conductivity of 3D printed porous ceramics. Reproduced with permission.^[^ ^116^ ^]^ Copyright 2022, Elsevier. g) Reversible hydrogen‐bond crosslink in inks, h) photographs of dual‐channel coextrusion 3D printing processes, and i) Butane flame combustion tests. Reproduced with permission.^[^ ^117^ ^]^ Copyright 2023, Elsevier.

Wang et al.^[^ ^115^ ^]^ introduced a versatile heat‐cured DIW approach for producing silica, alumina‐silica, and titanium dioxide‐silica heat‐resistant ceramic aerogels (**Figure** **8**a). In contrast to conventional silica aerogels, 3D printed ceramic aerogels exhibit exceptional stability at high temperatures in air, withstanding up to 1000 °C (experiencing linear shrinkage of less than 5%) (Figure 8b). This enhanced thermal resistance is attributed to the presence of a refractory fumed silica phase, which mitigates microstructural degradation of the ceramic aerogel at elevated temperatures. Benefiting from their low density (0.21 g cm^−3^), high surface area (284 m^2^ g^−1^), and evenly distributed mesopores, the 3D printed ceramic aerogels possess low thermal conductivity (30.87 mW m^−1^ K^−1^) (Figure 8c). Cao et al.^[^ ^116^ ^]^ developed a mullite fiber‐based porous ceramic using DLP, featuring complex geometries and exceptional high‐temperature thermal insulation properties. Uniform dispersion and structural stability of the mullite fibers were achieved through the use of photosensitive hydroxysiloxanes (HPMS‐KH570) as the resin matrix (Figure 8d). Optimal performance was observed when the fiber aspect ratio was 45 and the volume content was 6.67% (Figure 8e), resulting in a material with a density of 0.47 g cm^−3^ and a thermal conductivity of 0.11 W m^−1^ k^−1^ (Figure 8f). Remarkably, the material maintained stable thermal insulation performance at high temperatures ranging from 1000 to 1400 °C, with no significant increase in thermal conductivity even at 1200 °C. This low thermal conductivity under extreme conditions underscores its enhanced thermal insulation capabilities in high‐temperature environments, highlighting its promising potential for thermal insulation applications. Wang et al.^[^ ^117^ ^]^ introduced an innovative dual‐channel co‐extrusion printing method for crafting polyimide‐silica aerogels (Figure 8g,h), wherein the microscopic composite nature of polyimide and silica yielded enhanced mechanical properties (specific modulus: 54.45 kN m kg^−1^), notable flame retardancy (LOI value: 55.3%), and exceptional thermal stability (linear shrinkage: less than 5% at 400 °C). Additionally, the aerogels exhibited nanoporous characteristics including low density (0.196 g cm^−3^), high specific surface area (518 m^2^ g^−1^), and a concentrated pore size distribution (≈30 nm), resulting in a low thermal conductivity of 20.80 mW m^−1^ K^−1^, thus demonstrating significant potential for thermal insulation in medium to high‐temperature environments (Figure 8i).

#### 4.2.2 Silicon Carbide (SiC)

The aforementioned oxide ceramic aerosols like silicon oxide, zirconium oxide, and mullite face limitations in extreme high‐temperature conditions and rapid temperature fluctuations due to issues such as brittle fracture induced by grain coarsening, intergranular coefficient of thermal expansion mismatch, and low operational thresholds (<1300 °C).^[^ ^136^ ^]^ In contrast, silicon carbide, characterized by Si and C atoms arranged tetrahedrally, renders silicon carbide aerogels exceptionally promising as thermal protection materials in extreme environments, owing to their distinctive structural stability, low coefficient of thermal expansion, extraordinary temperature resistance (>1300 °C), corrosion resistance, and high oxidation resistance.^[^ ^137^ ^]^

**Figure 9:**
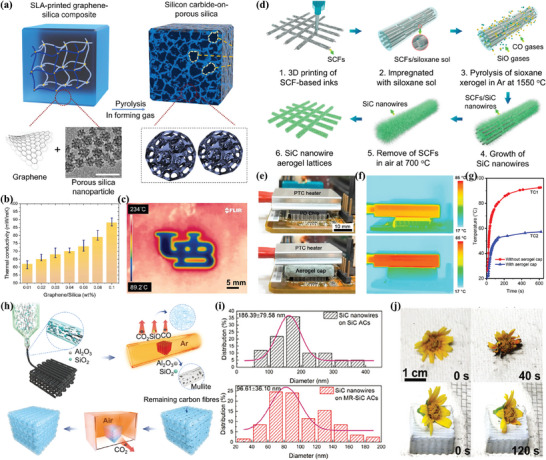
3D Printed Porous Silicon Carbide Ceramics for Thermal Insulation. a) Graphene and mesoporous silica are used as feedstock materials for DLP, b) thermal conductivity measurements of 3D‐printed samples, and c) an infrared (IR) image of a 3D‐printed logo placed on a hot plate. Reproduced with permission.^[^ ^119^ ^]^ Copyright 2022, Elsevier. d) Schematic illustrating the fabrication of SiC via DIW, e‐g) thermal management demonstration on the I/O chip of a computer motherboard. Reproduced with permission.^[^ ^122^ ^]^ Copyright 2022, American Chemical Society. h) Fabrication progress of 3D‐printed mullite‐reinforced SiC aerogels, i) average diameter of SiC nanowires, and j) morphological changes over time of a fresh flower placed on an asbestos wire net with burning alcohol flames without and with mullite‐reinforced SiC aerogels in between. Reproduced with permission.^[^ ^123^ ^]^ Copyright 2024, John Wiley and Sons.

Guo et al.^[^ ^119^ ^]^ utilized vat photopolymerization 3D printing for the production of structured conductive SiC ceramics characterized by both high electrical conductivity and low thermal conductivity, exhibiting electrical reliability at elevated temperatures surpassing 600 °C (**Figure** **9**a–c). The ceramics exhibited bulk densities ranging from 0.366 to 0.897 g cm^−3^ and thermal conductivities ranging from 62 to 88 mW m^−1^ K^−1^. Furthermore, the mechanical properties of the thermal insulation ceramics can be augmented through microstructural densification achieved via spark plasma sintering. In addition, Guo et al.^[^ ^122^ ^]^ employed DIW to craft elastic SiC nanowire aerogels featuring 3D structures (Figure 9d), allowing for programmable geometries and customizable mechanical properties. The Young's modulus of the resulting SiC nanowire aerogel lattice can be deliberately adjusted within a broad range spanning from 0.012 to 5.800 MPa, encompassing over two orders of magnitude. Notably, these engineered lightweight elastic silicon carbide nanowire aerogels exhibit a low thermal conductivity of 0.046 W m^−1^ K^−1^, making them ideal for use in electronic devices that require thermal insulation (Figure 9e–g).

However, there are significant challenges in translating theoretical research on SiC ceramics into practical applications. The primary issue lies in silicon carbide's inherent brittleness; despite its high hardness and wear resistance, it is prone to fracture under impact or bending loads, which restricts its application scope.^[^ ^136^ ^]^ To advance the development and utilization of SiC ceramics, it is crucial to enhance their structural integrity and simplify the production process, all while maintaining their excellent thermal insulation properties. Miao et al.^[^ ^123^ ^]^ developed composite structures containing carbon fibers, silica, and alumina precursors through DIW, followed by in situ growth of SiC nanowires during high‐temperature sintering (Figure 9h). This process resulted in mullite‐reinforced SiC aerogels with superior mechanical properties and exceptional thermal insulation. The material exhibits a multilayered, highly porous structure (90.0% porosity), where the incorporation of mullite not only facilitates the growth of smaller, longer SiC nanowires but also enhances mechanical strength and thermal stability (Figure 9i,j). This leads to a high Young's modulus (24.4 MPa) and compressive strength (1.65 MPa) while maintaining an ultra‐low thermal conductivity of 0.021 W m^−1^ k^−1^. The aerogel remains stable at temperatures up to 1200 °C, making it an excellent candidate for advanced thermal management in electronic devices and demonstrating its potential for high‐temperature insulation and structural reinforcement in the green energy era.

## 5 Challenges and Prospectives

Despite the significant advancements and achievements attained thus far, 3D printing of porous ceramics is still in its early stages and faces several challenges related to manufacturing quality, efficiency, and cost reduction. First, the formulation of ceramic powders and pastes for 3D printing must be precisely optimized to achieve desired properties such as porosity, thermal insulation, and mechanical strength, making the development and sourcing of these materials both complex and costly.^[^ ^138^ ^]^ Additionally, 3D printed porous ceramics often undergo sintering or curing processes that introduce shrinkage and stress, posing significant challenges in maintaining consistent pore structures and minimizing defects like cracks or irregular pore shapes.^[^ ^139^ ^]^ Furthermore, ceramic precursors tailored for 3D printing are typically more expensive than those used in traditional manufacturing methods. Addressing these challenges will require advances in materials science to develop low‐cost, high‐performance precursors, alongside innovations in 3D printing hardware and software to enhance precision and efficiency. Moreover, energy‐efficient post‐processing technologies will be critical to ensuring high quality while improving economic viability.

**Figure 10:**
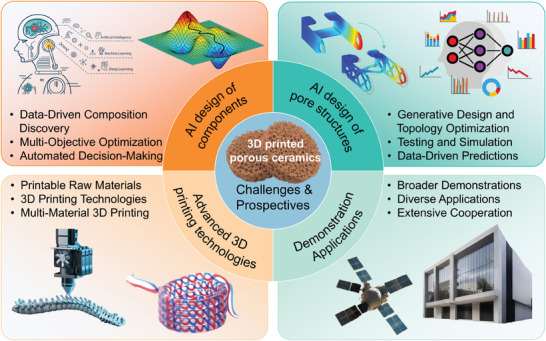
Challenges and prospectives of 3D printed porous ceramics. Reproduced with permission.^[^ ^141^, ^142^, ^143^ ^]^ Copyright 2022, Informa UK Limited; Copyright 2024, Elsevier; Copyright 2023, Springer Nature.

Therefore, there is a pressing need to comprehensively explore novel materials, innovative structures, and advanced processes of porous thermal insulation ceramics, while urgently developing integrated and optimized intelligent design approaches and high‐efficient manufacturing technologies that encompass the composition, structure, and performance aspects. Artificial intelligence (AI) holds significant potential to address these needs effectively,^[^ ^140^ ^]^ but several key challenges must be addressed. These include the development of AI‐driven approaches for optimizing the composition of thermal insulation ceramics, the implementation of intelligent strategies for designing pore structures, the establishment of high‐quality additive manufacturing technology, and the broadening of demonstration applications for porous thermal insulation ceramics in diverse settings (**Figure** **10**).

### 5.1 AI Design of Thermal Insulation Ceramic Components

The thermal insulation properties of ceramic materials are significantly influenced by their chemical composition, impacting key factors including thermal conductivity, heat capacity, and resistance to thermal degradation, all pivotal for effective insulation. For instance, metal oxide ceramics like Al_2_O_3_ and ZrO_2_ possess crystalline and bonded structures that effectively mitigate thermal conductivity, thus reducing heat transfer within the material.^[^ ^144^ ^]^ Composite oxide ceramics can incorporate the advantageous properties of diverse compositions, offering tailored thermal insulation performance to suit specific application needs.^[^ ^145^ ^]^ Consequently, the chemical composition of ceramic materials stands as a critical determinant of their thermal insulation capabilities, underscoring its importance in material design and selection for thermal insulation purposes.

Future research could explore the development of hybrid ceramic composites, where nano‐additives, such as graphene and aerogel, are integrated into the ceramic matrix to enhance thermal insulation and mechanical properties.^[^ ^146^ ^]^ New ceramic materials, including carbide‐based or nitride‐based ceramics, may be investigated for applications that demand extreme thermal stability. The incorporation of functional additives, such as phase change materials or reflective particles, could further improve thermal regulation and reflectivity.^[^ ^147^ ^]^ Moreover, the use of environmentally friendly raw materials, including biobased ceramics and recycled industrial by‐products, presents an opportunity to align material development with sustainable practices, supporting broader sustainability goals.

AI has fundamentally reshaped the approach to materials design, including thermal insulation ceramics. By employing AI‐driven computational models and sophisticated algorithms, extensive chemical spaces can be explored, numerous candidate materials rapidly generated,^[^ ^148^ ^]^ and the thermal insulation properties of novel ceramic compositions predicted with exceptional precision and efficiency. AI algorithms analyze vast datasets encompassing diverse material properties, chemical structures, and performance characteristics, uncovering intricate correlations beyond human comprehension. They also enable the identification of material compositions tailored to specific insulation requirements, such as high‐temperature resilience, low thermal conductivity, or resistance to thermal degradation. Moreover, AI incorporates factors like cost, raw material availability, and environmental impact into the design process, ensuring that the resulting materials excel in performance while remaining economically viable and sustainable.

### 5.2 AI Design of Pore Structures in Porous Thermal Insulation Ceramics

The pore structure of porous ceramics plays a pivotal role in determining thermal insulation performance. The size, shape, distribution, and spatial arrangement of pores significantly influence the material's ability to impede thermal transfer. Future research could focus on optimizing pore distribution by tuning the pore structure to disrupt thermal conduction and convection pathways, thereby enhancing thermal insulation performance while minimizing material usage.^[^ ^13^ ^]^ For example, exploring multiscale layered porous structures could offer tailored insulation solutions for specific thermal applications, while preserving mechanical strength.

AI has elevated the design of pore structures in porous ceramics to a higher level of sophistication.^[^ ^102^ ^]^ Traditional forward design, reliant on a priori knowledge and iterative trial‐and‐error methods, often results in prolonged design cycles and struggles to identify optimal configurations through heuristic approaches.^[^ ^149^ ^]^ Machine learning and other intelligent algorithms significantly accelerate the optimization process, expanding the search space for structural solutions. By training agent models, AI can establish mapping relationships between pore structures and thermal insulation performance, enabling the rapid, intelligent, and precise generation of target structures that meet specific performance requirements.

### 5.3 Advanced 3D Printing Technologies of Porous Thermal Insulation Ceramics

Rapid advancements in 3D printing enable precise control over the pore size, shape, distribution, and interconnectivity of porous ceramics at both millimeter, micron, and nano scales. This breakthrough significantly enhances structural design freedom, offering unparalleled flexibility and precision in fabricating complex porous ceramic architectures. Furthermore, multi‐material 3D printing allows seamless integration of diverse materials into porous structures, enabling the incorporation of additives, reinforcements, or phase change materials to further optimize thermal performance.^[^ ^150^ ^]^

Ceramic 3D printing currently lags behind its metal and polymer counterparts due to a limited range of available technologies, highlighting the need to expand the variety of printable raw materials and refine printing methodologies.^[^ ^139^ ^]^ The formability, defect sensitivity (e.g., cracks), and overall performance of 3D printed porous ceramics are influenced by fundamental factors, including raw material properties and 3D printing process variables.^[^ ^151^ ^]^ Optimizing the production of high‐performance ceramics requires a thorough understanding of the complex interactions between processing factors and material characteristics. Enhancing the correlation between 3D printing process parameters and material properties is critical for identifying optimal operational conditions. Future research should focus on expanding the range of printable materials, improving printing techniques, and developing processes capable of fabricating functionally graded structures through the integration of multiple ceramic components. Furthermore, advancing sintering and heat treatment techniques to minimize shrinkage and achieve precise porosity control will be essential for enhancing the performance of 3D printed porous ceramics.

Ensuring structural integrity and scalability for industrial production is paramount for 3D printed porous ceramics. Layer adhesion serves as a foundational factor directly impacting the reliability of printed porous ceramics.^[^ ^152^ ^]^ Precise control over printing parameters, such as exposure time and layer thickness, significantly enhances layer adhesion, mitigating risks of delamination and cracking. This is crucial for scaling up production while maintaining consistent product quality. Additionally, shrinkage control during sintering or post‐processing is critical, as excessive shrinkage often results in dimensional inaccuracies, internal stresses, and cracks, which not only hinder scalability but also compromise product consistency and tolerance requirements.^[^ ^88^ ^]^ Addressing this challenge necessitates meticulous regulation of sintering parameters, including temperature and duration. Simultaneously, porosity control is vital for achieving the desired functional performance, as variations in pore size, distribution, or interconnectivity can adversely affect both performance and structural reliability. Advanced monitoring and quality assurance systems are therefore indispensable to ensure the reproducibility of production batches and maintain consistent porosity.^[^ ^153^ ^]^ These measures collectively underpin the successful commercialization of 3D printed porous ceramics, enabling reliable performance and scalable manufacturing.

### 5.4 Demonstration for Porous Thermal Insulation Ceramics

Porous thermal insulating ceramics hold significant potential for enhancing thermal insulation across a range of industries, yet their adoption remains limited. To fully realize their potential, it is essential to expand their application beyond traditional sectors like aerospace and construction into emerging fields such as renewable energy, automotive, and healthcare. In renewable energy, these ceramics can enhance the efficiency and durability of solar panels and energy storage systems. In automotive manufacturing, they contribute to lightweight designs and optimized thermal management in vehicles.^[^ ^154^ ^]^ Additionally, in healthcare, porous insulating ceramics offer opportunities to advance medical devices and implants with improved biocompatibility and thermal resilience.^[^ ^155^ ^]^ Proactive demonstration of their utility across these diverse applications will foster innovation and broaden their use in addressing modern industry's evolving needs. Future research should prioritize the development of responsive ceramics capable of adapting their thermal insulation performance to temperature fluctuations or other external conditions. Investigating the long‐term performance of porous ceramics under varying environmental factors, such as thermal cycling, humidity, and mechanical stress, will be critical for improving their service life and providing valuable insights to guide ceramic design.

## 6 Conclusion

Porous ceramics exhibit significant potential in thermal insulation applications, where the design of pore structures plays a critical role due to the impact of thermal convection, radiation, and conduction mechanisms within porous materials. Conventional fabrication techniques, such as partial sintering, freeze‐drying, direct foaming, and sacrificial templating, face inherent limitations in achieving highly customized and precise pore structures. The advent of 3D printing has revolutionized the field, enabling computational modeling for design optimization and providing precise control over porosity and structural parameters through the fine‐tuning of processing conditions. Currently, 3D printed porous ceramics predominantly include metal oxides (e.g., Al_2_O_3_, ZrO_2_) and non‐metallic oxides and carbides (e.g., SiO_2_, SiC), which have shown promising advancements in thermal insulation applications. However, compared to 3D printing of porous metals and polymers, the 3D printing of porous ceramics remains in its early stages. Further research is required to explore new ceramic materials, develop innovative porous structures, and advanced ceramic 3D printing technologies. The integration of AI offers substantial promise in developing intelligent design methods for optimizing material composition and porous structures, as well as for advancing 3D printing technologies. Additionally, expanding the practical applications of porous thermal insulation ceramics across various industries is essential to fully unlock their potential and address the increasingly complex challenges of modern engineering.

## Conflict of Interest

The authors declare no conflict of interest.

## References

[1] Y. Ding , C. H. Dreimol , R. Zboray , K. Tu , S. Stucki , T. Keplinger , G. Panzarasa , I. Burgert , Mate. Horiz. 2023, 10, 257. 10.1039/d2mh01016j PMC9810104 36409220

[2] G. P. Thiel , A. K. Stark , Joule. 2021, 5, 531.

[3] A. Rode , T. Carleton , M. Delgado , M. Greenstone , T. Houser , S. Hsiang , A. Hultgren , A. Jina , R. E. Kopp , K. E. McCusker , I. Nath , J. Rising , J. Yuan , Nature. 2021, 598, 308. 34646000 10.1038/s41586-021-03883-8

[4] L. T. Biardeau , L. W. Davis , P. Gertler , C. Wolfram , Nat. Sustainable. 2020, 3, 25.

[5] V. Apostolopoulou‐Kalkavoura , P. Munier , L. Bergström , Adv. Mater. 2021, 33, 2001839. 32761673 10.1002/adma.202001839 PMC11468958

[6] S. Ahankari , P. Paliwal , A. Subhedar , H. Kargarzadeh , ACS Nano. 2021, 15, 3849. 33710860 10.1021/acsnano.0c09678

[7] J. Guo , S. Fu , Y. Deng , X. Xu , S. Laima , D. Liu , P. Zhang , J. Zhou , H. Zhao , H. Yu , S. Dang , J. Zhang , Y. Zhao , H. Li , X. Duan , Nature. 2022, 606, 909. 35768591 10.1038/s41586-022-04784-0 PMC9242853

[8] C. Jia , L. Li , Y. Liu , B. Fang , H. Ding , J. Song , Y. Liu , K. Xiang , S. Lin , Z. Li , W. Si , B. Li , X. Sheng , D. Wang , X. Wei , H. Wu , Nat. Commun. 2020, 11, 3732. 32709868 10.1038/s41467-020-17533-6 PMC7382455

[9] L. An , J. Wang , D. Petit , J. N. Armstrong , K. Hanson , J. Hamilton , M. Souza , D. Zhao , C. Li , Y. Liu , Nano Lett. 2020, 20, 3828. 32267711 10.1021/acs.nanolett.0c00917

[10] C. Li , Y. Yang , G. Xu , Y. Zhou , M. Jia , S. Zhong , Y. Gao , C. Park , Q. Liu , Y. Wang , High Voltage. 2022, 7, 610.

[11] X. Cheng , Y. T. Liu , Y. Si , J. Yu , B. Ding , Nat. Commun. 2022, 13, 2637. 35552405 10.1038/s41467-022-30435-z PMC9098874

[12] S. Sobhani , S. Allan , P. Muhunthan , E. Boigne , M. Ihme , Adv. Eng. Mater. 2020, 22, 2000158.

[13] Y. Chen , N. Wang , O. Ola , Y. Xia , Y. Zhu , Mater. Sci. Eng. R: Rep. 2021, 143, 100589.

[14] X. Zhang , W. Huo , J. Liu , Y. Zhang , S. Zhang , J. Yang , J. Eur. Ceram. Soc. 2020, 40, 930.

[15] F. Zhang , Z. Li , M. Xu , S. Wang , N. Li , J. Yang , J. Eur. Ceram. Soc. 2022, 42, 3351.

[16] K. Jiang , M. Xia , Y. Tang , Y. Xu , T. Deng , B. Li , W. Chen , J. Eur. Ceram. Soc. 2023, 43, 1689.

[17] C. Vakifahmetoglu , T. Semerci , G. D. Soraru , J. Am. Ceram. Soc. 2020, 103, 2941.

[18] Y. Dong , X. Dong , L. Li , J. Wu , L. Yan , J. Liu , A. Guo , Ceram. Int. 2021, 47, 21029.

[19] B. L. Hao , Y. Lang , D. Q. Bian , C. A. Wang , J. Am. Ceram. Soc. 2020, 103, 4602.

[20] B. Dermeik , N. Travitzky , Adv. Eng. Mater. 2020, 22, 2000256.

[21] Y. Chen , W. Guo , Y. Luo , Z. Ma , L. Zhang , Z. Yue , J. Am. Ceram. Soc. 2021, 104, 5679.

[22] Z. Du , D. Yao , Y. Xia , K. Zuo , J. Yin , H. Liang , Y. P. Zeng , Ceram. Int. 2020, 46, 12942.

[23] S. M. Sajadi , L. Vásárhelyi , R. Mousavi , A. H. Rahmati , Z. Kónya , Á. Kukovecz , T. Arif , T. Filleter , R. Vajtai , P. Boul , Sci. Adv. 2021, 7, eabc5028. 34233870 10.1126/sciadv.abc5028 PMC8262818

[24] Y. Oh , V. Bharambe , B. Mummareddy , J. Martin , J. McKnight , M. A. Abraham , J. M. Walker , K. Rogers , B. Conner , P. Cortes , E. MacDonald , J. J. Adams , Addit. Manuf. 2019, 27, 586.

[25] C. Cai , W. S. Tey , J. Chen , W. Zhu , X. Liu , T. Liu , L. Zhao , K. Zhou , J. Mater. Process. Technol. 2021, 288, 116882.

[26] H. Chen , L. Guo , W. Zhu , C. Li , Polymers. 2022, 14, 4635. 36365628 10.3390/polym14214635 PMC9654317

[27] P. Ruckdeschel , A. Philipp , M. Retsch , Adv. Funct. Mater. 2017, 27, 1702256.

[28] Q. Sun , Z. Xue , Y. Chen , R. Xia , J. Wang , S. Xu , J. Zhang , Y. Yue , Int. J. Extreme. Manuf. 2022, 4, 015001.

[29] C. R. Doering , Proc. Natl. Acad. Sci. 2020, 117, 9671. 32345714 10.1073/pnas.2004239117 PMC7211936

[30] F. Hu , S. Wu , Y. Sun , Adv. Mater. 2019, 31, 1801001. 10.1002/adma.201801001 30379354

[31] K. J. Shayegan , S. Biswas , B. Zhao , S. Fan , H. A. Atwater , Nat. Photonics. 2023, 17, 891.

[32] H. A. Eivari , Z. Sohbatzadeh , P. Mele , M. Assadi , Mater. Today. Energy. 2021, 21, 100744.

[33] H. Babaei , C. E. Wilmer , Phys. Rev. Lett. 2016, 116, 025902. 26824553 10.1103/PhysRevLett.116.025902

[34] K. Su , Y. Wang , K. Hu , X. Fang , J. Yao , Q. Li , J. Yang , ACS Appl. Mater. Interfaces. 2021, 13, 22017. 33909396 10.1021/acsami.1c03543

[35] M. Luo , C. Wang , J. Zhao , L. Liu , Int. J. Heat Mass Transfer. 2022, 188, 122597.

[36] Y. Zhang , J. Materiomics. 2016, 2, 237.

[37] C. Wan , Y. Wang , W. Norimatsu , M. Kusunoki , K. Koumoto , Appl. Phys. Lett. 2012, 100, 101913.

[38] W. X. Zhou , Y. Cheng , K. Q. Chen , G. Xie , T. Wang , G. Zhang , Adv. Funct. Mater. 2020, 30, 1903829.

[39] S. S. Shrestha , J. Tiwari , A. Rai , D. E. Hun , D. Howard , A. O. Desjarlais , M. Francoeur , T. Feng , Int. J. Therm. Sci. 2023, 187, 108164.

[40] H. Yu , H. Zhang , P. Buahom , J. Liu , X. Xia , C. B. Park , Energy. 2021, 233, 121140.

[41] J. H. Qiao , R. Bolot , H. L. Liao , C. Coddet , J. Therm. Spray Technol. 2013, 22, 175.

[42] J. Qian , H. Wu , F. Wang , Appl. Surf. Sci. 2023, 611, 155613.

[43] Z. Guo , R. Wang , K. Xu , PhRvE. 2015, 91, 033313. 10.1103/PhysRevE.91.033313 25871252

[44] B. E. Rapp , in Microfluidics: Modelling, Mechanics and Mathematics, (Ed: B. E. Rapp ), Elsevier, Amsterdam, New York 2017, 243–263.

[45] S. Rashidi , J. A. Esfahani , N. Karimi , Renewable. Sustainable. Energy. Rev. 2018, 91, 229.

[46] L. Qiu , H. Zou , D. Tang , D. Wen , Y. Feng , X. Zhang , Appl. Therm. Eng. 2018, 130, 1004.

[47] G. Hayase , K. Kugimiya , M. Ogawa , Y. Kodera , K. Kanamori , K. Nakanishi , J. Mater. Chem. A. 2014, 2, 6525.

[48] A. G. M. Pornea , J. M. C. Puguan , J. L. A. Ruello , H. Kim , ACS Appl. Polym. Mater. 2022, 4, 2880.

[49] N. Yan , Q. Fu , M. Tong , J. Zhang , J. Sun , Q. Shen , Compos. B: Eng. 2021, 222, 109040.

[50] J. Feng , B. L. Su , H. Xia , S. Zhao , C. Gao , L. Wang , O. Ogbeide , J. Feng , T. Hasan , Chem. Soc. Rev. 2021, 50, 3842. 33522550 10.1039/c9cs00757a

[51] Y. Man , G. Ding , L. Xudong , K. Xue , D. Qu , Z. Xie , J. Asian Ceram. Soc. 2021, 9, 1377.

[52] X. Wang , X. Wang , X. Niu , X. Qiu , L. Wang , Int. J. Thermophys. 2020, 41, 145.

[53] P. Šimonová , W. Pabst , J. Am. Ceram. Soc. 2024, 107, 1262.

[54] S. Rajpoot , J. H. Ha , Y. W. Kim , Ceram. Int. 2021, 47, 8668.

[55] S. Wang , Y. Yang , J. Cui , X. Liu , S. Zhang , Q. Jia , Ceram. Int. 2022, 48, 27051.

[56] S. M. Miller , X. Xiao , K. T. Faber , J. Eur. Ceram. Soc. 2015, 35, 3595.

[57] L. Yuan , E. Jin , C. Li , Z. Liu , C. Tian , B. Ma , J. Yu , Ceram. Int. 2021, 47, 9017.

[58] M. Sun , S. Yang , X. Gao , P. Man , J. Qu , W. Zhang , S. Yin , L. Cheng , Ceram. Int. 2021, 47, 8169.

[59] F. Xia , S. Cui , X. Pu , Ceram. Int. 2022, 48, 5197.

[60] J. Zhao , X. Ban , Y. Yang , Z. Yuan , H. Ru , D. Su , Materials. 2023, 16, 1342. 36836972 10.3390/ma16041342 PMC9962626

[61] J. Qiao , Y. Wen , Ceram. Int. 2020, 46, 1442.

[62] H. Zhang , H. Liu , M. Zhu , H. Wu , M. Yuan , X. Liu , Z. Huang , Ceram. Int. 2023, 49, 27604.

[63] P. C. Ho , H. K. Chang , P.‐Y. Chen , J. Am. Ceram. Soc. 2024, 107, 7550.

[64] R. K. Bordia , S. J. L. Kang , E. A. Olevsky , J. Am. Ceram. Soc. 2017, 100, 2314.

[65] S. Pinches , G. V. Franks , J. Am. Ceram. Soc. 2023, 106, 5167.

[66] Y. Chen , J. Sun , P. Jiang , Z. Chai , B. Zhang , J. Li , ChemBioEng Rev. 2023, 10, 941.

[67] Y. C. Gao , B. Qin , S. M. Wen , Y. You , J. Xue , Y. C. Yin , Z. Y. Ma , K. Dong , Y. F. Meng , I. Manke , S. C. Zhang , Z. L. Yu , S. H. Yu , Nano Lett. 2023, 23, 9011. 37676743 10.1021/acs.nanolett.3c02654

[68] J. H. Shepherd , R. J. Friederichs , S. M. Best , in Hydroxyapatite (Hap) for Biomedical Applications, (Ed: M. Mucalo ), Woodhead Publishing, England 2015, pp. 235–267.

[69] N. Galvani , M. Pasquet , A. Mukherjee , A. Requier , S. Cohen‐Addad , O. Pitois , R. Höhler , E. Rio , A. Salonen , D. J. Durian , D. Langevin , Proc. Natl. Acad. Sci. 2023, 120, e2306551120. 37708201 10.1073/pnas.2306551120 PMC10515135

[70] M. Mao , Z. Meng , X. Huang , H. Zhu , L. Wang , X. Tian , J. He , D. Li , B. Lu , Int. J. Extreme Manuf. 2024, 6, 023001.

[71] X. Li , J. W. Chua , X. Yu , Z. Li , M. Zhao , Z. Wang , W. Zhai , Adv. Sci. 2024, 11, 2305232. 10.1002/advs.202305232 PMC10939082 37997188

[72] Y. Gao , J. Lalevée , A. Simon‐Masseron , Adv. Mater. Technol. 2023, 8, 2300377.

[73] R. D. Weeks , R. L. Truby , S. G. M. Uzel , J. A. Lewis , Adv. Mater. 2023, 35, 2206958. 10.1002/adma.202206958 36404106

[74] R. Karyappa , D. Zhang , Z. Qiang , J. Rong , A. Suwardi , H. Liu , Addit. Manuf. 2024, 79, 103903.

[75] M. Li , S. Huang , E. Willems , J. Soete , M. Inokoshi , B. Van Meerbeek , J. Vleugels , F. Zhang , Adv. Mater. 2024, 36, 2306764. 10.1002/adma.202306764 37986661

[76] M. Cheype , V. Pateloup , S. Bernard , Adv. Mater. 2024, 36, 2307554. 10.1002/adma.202307554 37906971

[77] G. Qi , L. Zhiqin , W. Zhaolong , K. Kavin , Z. Wang , H. Xiangnan , Z. Jianlin , X. F. Nicholas , Int. J. Extreme Manuf. 2020, 2, 022004.

[78] S. Zakeri , M. Vippola , E. Levänen , Addit. Manuf. 2020, 35, 101177.

[79] S. O'Halloran , A. Pandit , A. Heise , A. Kellett , Adv. Sc. 2023, 10, 2204072. 10.1002/advs.202204072 PMC9982557 36585380

[80] J. C. Sänger , B. R. Pauw , B. Riechers , A. Zocca , J. Rosalie , R. Maaß , H. Sturm , J. Günster , Adv. Mater. 2023, 35, 2208653. 10.1002/adma.202208653 36445940

[81] B. Weidinger , G. Yang , N. von Coelln , H. Nirschl , I. Wacker , P. Tegeder , R. R. Schröder , E. Blasco , Adv. Sci. 2023, 10, 2302756. 10.1002/advs.202302756 PMC10558687 37532671

[82] I. Gibson , D. Rosen , B. Stucker , M. Khorasani , I. Gibson , D. Rosen , B. Stucker , M. Khorasani , Additive Manufacturing Technologies, (Ed: I. Gibson , D. Rosen , B. Stucker , M. Khorasani ), Springer, Cham, Switzerland 2021, 237–252.

[83] M. Kwon , J. H. Choi , J. H. Kim , J. H. Choi , U. S. Kim , K. T. Hwang , Y. M. Kang , K. S. Han , Addit. Manuf. 2023, 70, 103564.

[84] H. Sun , B. Zou , X. Wang , W. Chen , G. Zhang , T. Quan , C. Huang , Mater. Chem. Phys. 2024, 319, 129337.

[85] S. Zhong , Q. Shi , Y. Deng , Y. Sun , C. Politis , S. Yang , Ceram. Int. 2022, 48, 33485.

[86] E. Willems , M. Turon‐Vinas , B. C. Dos Santos , B. Van Hooreweder , F. Zhang , B. Van Meerbeek , J. Vleugels , J. Eur. Ceram. Soc. 2021, 41, 5292.

[87] A. M. Wätjen , P. Gingter , M. Kramer , R. Telle , Adv. Mech. Eng. 2014, 6, 141346.

[88] Y. Zhao , J. Zhu , W. He , Y. Liu , X. Sang , R. Liu , Nat. Commun. 2023, 14, 2381. 37185359 10.1038/s41467-023-38082-8 PMC10130026

[89] N. Xu , X. Ye , D. Wei , J. Zhong , Y. Chen , G. Xu , D. He , ACS Appl. Mater. Interfaces. 2014, 6, 14952. 25133309 10.1021/am502716t

[90] H. Wang , L. Y. Liu , P. Ye , Z. Huang , A. Y. R. Ng , Z. Du , Z. Dong , D. Tang , C. L. Gan , Adv. Mater. 2021, 33, 2007072. 10.1002/adma.202007072 33682251

[91] Y. Sun , M. Li , Y. Jiang , B. Xing , M. Shen , C. Cao , C. Wang , Z. Zhao , Adv. Eng. Mater. 2021, 23, 2001475.

[92] J. Bauer , C. Crook , A. Guell Izard , Z. C. Eckel , N. Ruvalcaba , T. A. Schaedler , L. Valdevit , Matter. 2019, 1, 1547.

[93] Z. Yang , L. Yang , P. Wang , Z. Peng , Y. Niu , W. Jiang , Z. Fan , Addit. Manuf. 2024, 79, 103898.

[94] S. S. L. Chan , R. M. Pennings , L. Edwards , G. V. Franks , Addit. Manuf. 2020, 35, 101335.

[95] Z. Liu , Q. Lei , S. Xing , J. Mater. Res. Technol. 2019, 8, 3741.

[96] X. Liu , B. Zou , H. Xing , C. Huang , Ceram. Int. 2020, 46, 937.

[97] Y. Ye , Y. Du , T. Hu , J. You , B. Bao , Y. Wang , T. Wang , Ind. Eng. Chem. Res. 2021, 60, 9368.

[98] I. Cooperstein , S. R. K. C. Indukuri , A. Bouketov , U. Levy , S. Magdassi , Adv. Mater. 2020, 32, 2001675. 10.1002/adma.202001675 32419262

[99] M. Mariani , R. Beltrami , P. Brusa , C. Galassi , R. Ardito , N. Lecis , J. Eur. Ceram. Soc. 2021, 41, 5307.

[100] J. Yang , X. Shen , W. Yang , J. K. Kim , Prog. Mater. Sci. 2023, 133, 101054.

[101] A. Azizi , M. Ghassemi , High Voltage. 2024, 8, 1.

[102] C. S. Ha , D. Yao , Z. Xu , C. Liu , H. Liu , D. Elkins , M. Kile , V. Deshpande , Z. Kong , M. Bauchy , X. Zheng , Nat. Commun. 2023, 14, 5765. 37718343 10.1038/s41467-023-40854-1 PMC10505607

[103] P. Liu , Z. Han , W. Wu , Y. Zhao , Y. Song , M. Chai , Int. J. Heat Mass Transfer. 2024, 225, 125428.

[104] M. Saadi , A. Maguire , N. T. Pottackal , M. S. H. Thakur , M. M. Ikram , A. J. Hart , P. M. Ajayan , M. M. Rahman , Adv. Mater. 2022, 34, 2108855. 10.1002/adma.202108855 35246886

[105] P. Czyżewski , D. Marciniak , B. Nowinka , M. Borowiak , M. Bieliński , Polymers. 2022, 14, 356. 35054759 10.3390/polym14020356 PMC8779709

[106] J. Nomani , D. Wilson , M. Paulino , M. I. Mohammed , Mater. Today Commun. 2020, 22, 100626.

[107] M. R. Khosravani , F. Berto , M. R. Ayatollahi , T. Reinicke , Sci. Rep. 2022, 12, 1016. 35046490 10.1038/s41598-022-05005-4 PMC8770637

[108] X. Xu , S. Fu , J. Guo , H. Li , Y. Huang , X. Duan , Mater. Today. 2021, 42, 162.

[109] G. Yang , R. Guan , H. Zhen , K. Ou , J. Fang , D.‐s. Li , Q. Fu , Y. Sun , ACS Appl. Mater. Interfaces. 2022, 14, 10998. 35188368 10.1021/acsami.1c24090

[110] C. Yang , W. Wu , Z. Fu , H. Zheng , J. Mater. Sci. 2023, 58, 11992.

[111] S. Zhao , G. Siqueira , S. Drdova , D. Norris , C. Ubert , A. Bonnin , S. Galmarini , M. Ganobjak , Z. Pan , S. Brunner , G. Nyström , J. Wang , M. M. Koebel , W. J. Malfait , Nature. 2020, 584, 387. 32814885 10.1038/s41586-020-2594-0

[112] L. Wang , J. Feng , Y. Luo , Z. Zhou , Y. Jiang , X. Luo , L. Xu , L. Li , J. Feng , ACS Appl. Mater. Interfaces. 2021, 13, 40964. 34424660 10.1021/acsami.1c12020

[113] L. An , Z. Guo , Z. Li , Y. Fu , Y. Hu , Y. Huang , F. Yao , C. Zhou , S. Ren , Nat. Commun. 2022, 13, 4309. 35879371 10.1038/s41467-022-32027-3 PMC9314391

[114] L. Wang , J. Feng , Y. Jiang , D. Lu , J. Men , Y. Luo , X. Wang , J. Feng , Chem. Eng. J. 2023, 455, 140818.

[115] L. Wang , J. Feng , Y. Luo , Y. Jiang , G. Zhang , J. Feng , Small Methods. 2022, 6, 2200045. 10.1002/smtd.202200045 35344287

[116] Y. Cao , X. Xu , Z. Qin , C. He , L. Yan , F. Hou , J. Liu , A. Guo , Addit. Manuf. 2022, 60, 103235.

[117] L. Wang , J. Feng , S. Zhang , Q. Sun , Y. Luo , J. Men , W. He , Y. Jiang , L. Li , J. Feng , Addit. Manuf. 2023, 71, 103583.

[118] A. Dutto , M. Zanini , E. Jeoffroy , E. Tervoort , S. A. Mhatre , Z. B. Seibold , M. Bechthold , A. R. Studart , Adv. Mater. Technol. 2023, 8, 2201109.

[119] Z. Guo , L. An , S. Khuje , A. Chivate , J. Li , Y. Wu , Y. Hu , J. Armstrong , S. Ren , C. Zhou , Addit. Manuf. 2022, 59, 103109.

[120] W. Du , W. Yu , D. M. France , M. Singh , D. Singh , Sol. Energy. 2022, 236, 654.

[121] D. Tang , S. Xu , K. Yang , T. Gao , H. Tang , Ceram. Int. 2024, 50, 10618.

[122] P. Guo , L. Su , K. Peng , D. Lu , L. Xu , M. Li , H. Wang , ACS Nano. 2022, 16, 6625. 35404589 10.1021/acsnano.2c01039

[123] M. Miao , J. Yin , Z. Mao , Y. Chen , J. Lu , Small. 2024, 20, 2401742. 10.1002/smll.202401742 38721985

[124] Z. Zhang , J. Li , Y. Shi , X. Gu , S. Wang , R. Yang , L. Cao , X. Zhang , Mater. Today Adv. 2024, 21, 100466.

[125] M. Y. Moshkovitz , D. Paz , S. Magdassi , Adv. Mater. Technol. 2023, 8, 2300123.

[126] Y. Liu , Y. Liu , W. She , W. Li , Y. Cao , J. Wang , Ceram. Int. 2023, 49, 27514.

[127] Z. Gao , J. Yin , P. Liu , Q. Li , R. Zhang , H. Yang , H. Zhou , Int. J. Extreme Manuf. 2023, 5, 035001.

[128] X. Zhang , F. Wang , L. Dou , X. Cheng , Y. Si , J. Yu , B. Ding , ACS Nano. 2020, 14, 15616. 33118799 10.1021/acsnano.0c06423

[129] R. Wu , T. Zeng , M. Fan , Y. Cui , G. Xu , X. Wang , S. Cheng , Ceram. Int. 2023, 49, 33369.

[130] C. Jiao , J. Gu , Y. Cao , D. Xie , H. Liang , R. Chen , T. Shi , L. Shen , C. Wang , Z. Tian , X. Yi , J. Eur. Ceram. Soc. 2020, 40, 6087.

[131] X. Wen , B. Zhang , W. Wang , F. Ye , S. Yue , H. Guo , G. Gao , Y. Zhao , Q. Fang , C. Nguyen , X. Zhang , J. Bao , J. T. Robinson , P. M. Ajayan , J. Lou , Nat. Mater. 2021, 20, 1506. 34650230 10.1038/s41563-021-01111-2

[132] R. G. Martinez , E. Goiti , G. Reichenauer , S. Zhao , M. Koebel , A. Barrio , Energy Build. 2016, 128, 111.

[133] Y. Yu , L. Li , Y. Huang , L. Huang , S. Zhang , J. Non‐Cryst. Solids. 2022, 590, 121572.

[134] H. Pang , Z. Li , Int. J. Therm. Sci. 2021, 160, 106681.

[135] D. Jiang , J. Qin , X. Zhou , Q. Li , D. Yi , B. Wang , Ceram. Int. 2022, 48, 16290.

[136] X. Zhang , J. Yu , C. Zhao , Y. Si , Small. 2024, 20, 2311464. 10.1002/smll.202311464 38511588

[137] X. Wang , X. Gao , Z. Zhang , L. Cheng , H. Ma , W. Yang , J. Eur. Ceram. Soc. 2021, 41, 4671.

[138] Z. Chen , Z. Li , J. Li , C. Liu , C. Lao , Y. Fu , C. Liu , Y. Li , P. Wang , Y. He , J. Eur. Ceram. Soc. 2019, 39, 661.

[139] S. Bose , E. K. Akdogan , V. K. Balla , S. Ciliveri , P. Colombo , G. Franchin , N. Ku , P. Kushram , F. Niu , J. Pelz , A. Rosenberger , A. Safari , Z. Seeley , R. W. Trice , L. Vargas‐Gonzalez , J. P. Youngblood , A. Bandyopadhyay , J. Am. Ceram. Soc. 2024, 107, 7879.

[140] Z. Zhu , D. W. H. Ng , H. S. Park , M. C. McAlpine , Nat. Rev. Mater. 2021, 6, 27.

[141] B. Xue , Z. Wu , InFer. 2022, 226, 277.

[142] J. Yang , Y. Ma , C. Ma , J. Water Process Eng. 2024, 61, 105264.

[143] N. M. Larson , J. Mueller , A. Chortos , Z. S. Davidson , D. R. Clarke , J. A. Lewis , Nature. 2023, 613, 682. 36653452 10.1038/s41586-022-05490-7

[144] X. Dong , Q. An , S. Zhang , H. Yu , M. Wang , Ceram. Int. 2023, 49, 31035.

[145] A. Nisar , C. Zhang , B. Boesl , A. Agarwal , Ceram. Int. 2020, 46, 25845.

[146] S. Ahmad , S. Ali , M. Salman , A. H. Baluch , Ceram. Int. 2021, 47, 33956.

[147] A. Usman , F. Xiong , W. Aftab , M. Qin , R. Zou , Adv. Mater. 2022, 34, 2202457. 10.1002/adma.202202457 35616900

[148] R. Pollice , G. dos Passos Gomes , M. Aldeghi , R. J. Hickman , M. Krenn , C. Lavigne , M. Lindner‐D'Addario , A. Nigam , C. T. Ser , Z. Yao , A. Aspuru‐Guzik , Acc. Chem. Res. 2021, 54, 849. 33528245 10.1021/acs.accounts.0c00785 PMC7893702

[149] X. Zheng , X. Zhang , T. T. Chen , I. Watanabe , Adv. Mater. 2023, 35, 2302530. 10.1002/adma.202302530 37332101

[150] M. Rafiee , R. D. Farahani , D. Therriault , Adv. Sci. 2020, 7, 1902307. 10.1002/advs.201902307 PMC7312457 32596102

[151] Z. Ren , L. Gao , S. J. Clark , K. Fezzaa , P. Shevchenko , A. Choi , W. Everhart , A. D. Rollett , L. Chen , T. Sun , Science. 2023, 379, 89. 36603080 10.1126/science.add4667

[152] Y. Xin , X. Zhou , H. Bark , P. S. Lee , Adv. Mater. 2024, 36, 2307963. 10.1002/adma.202307963 37971199

[153] D. Guirguis , C. Tucker , J. Beuth , Nat. Commun. 2024, 15, 582. 38233405 10.1038/s41467-024-44783-5 PMC10794417

[154] C. L. Cramer , E. Ionescu , M. Graczyk‐Zajac , A. T. Nelson , Y. Katoh , J. J. Haslam , L. Wondraczek , T. G. Aguirre , S. LeBlanc , H. Wang , M. Masoudi , E. Tegeler , R. Riedel , P. Colombo , M. Minary‐Jolandan , J. Eur. Ceram. Soc. 2022, 42, 3049.

[155] J. L. Hernandez , K. A. Woodrow , Adv. Healthcare Mater. 2022, 11, 2102087.

